# Lentiviral Vectors and Protocols for Creation of Stable hESC Lines for Fluorescent Tracking and Drug Resistance Selection of Cardiomyocytes

**DOI:** 10.1371/journal.pone.0005046

**Published:** 2009-04-08

**Authors:** Hiroko Kita-Matsuo, Maria Barcova, Natalie Prigozhina, Nathan Salomonis, Karen Wei, Jeffrey G. Jacot, Brandon Nelson, Sean Spiering, René Haverslag, Changsung Kim, Maria Talantova, Ruchi Bajpai, Diego Calzolari, Alexey Terskikh, Andrew D. McCulloch, Jeffrey H. Price, Bruce R. Conklin, H. S. Vincent Chen, Mark Mercola

**Affiliations:** 1 Burnham Institute for Medical Research, La Jolla, California, United States of America; 2 Department of Bioengineering, University of California San Diego, La Jolla, California, United States of America; 3 Division of Cardiology, Department of Medicine, University of California San Diego, La Jolla, California, United States of America; 4 Gladstone Institute of Cardiovascular Disease, University of California San Francisco, San Francisco, California, United States of America; Ordway Research Institute, United States of America

## Abstract

**Background:**

Developmental, physiological and tissue engineering studies critical to the development of successful myocardial regeneration therapies require new ways to effectively visualize and isolate large numbers of fluorescently labeled, functional cardiomyocytes.

**Methodology/Principal Findings:**

Here we describe methods for the clonal expansion of engineered hESCs and make available a suite of lentiviral vectors for that combine Blasticidin, Neomycin and Puromycin resistance based drug selection of pure populations of stem cells and cardiomyocytes with ubiquitous or lineage-specific promoters that direct expression of fluorescent proteins to visualize and track cardiomyocytes and their progenitors. The phospho-glycerate kinase (PGK) promoter was used to ubiquitously direct expression of histone-2B fused eGFP and mCherry proteins to the nucleus to monitor DNA content and enable tracking of cell migration and lineage. Vectors with T/Brachyury and α-myosin heavy chain (αMHC) promoters targeted fluorescent or drug-resistance proteins to early mesoderm and cardiomyocytes. The drug selection protocol yielded 96% pure cardiomyocytes that could be cultured for over 4 months. Puromycin-selected cardiomyocytes exhibited a gene expression profile similar to that of adult human cardiomyocytes and generated force and action potentials consistent with normal fetal cardiomyocytes, documenting these parameters in hESC-derived cardiomyocytes and validating that the selected cells retained normal differentiation and function.

**Conclusion/Significance:**

The protocols, vectors and gene expression data comprise tools to enhance cardiomyocyte production for large-scale applications.

## Introduction

The minimal ability of the adult human heart to regenerate lost or damaged cardiomyocytes has led to an intense effort to direct human embryonic stem cells (hESCs) to form cardiomyocytes in order to model human heart disease and develop therapies [1]. hESC-derived cardiomyocytes resemble immature human fetal cardiomyocytes by multiple criteria, including electrophysiology [2], [3], calcium handling [3], [4], [5], force generation [5], and contractile protein expression and myofibrillar structure [6]. Since hESC-derived cardiomyocytes have the potential to engraft into surgical models of heart disease [7], [8], they have been considered for cardiomyocyte replacement therapy and as well as a tool to discover drugs capable of stimulating endogenous regeneration. Despite such encouraging advances, the application of hESC-derived cardiomyocytes for basic developmental research and large-scale applications, such as high throughput screening, toxicology testing and large animal studies, has been hindered by their poor yield from the heterogeneous hESC cultures and the difficulty of manipulating hESCs to express uniform levels of reporter constructs. We have therefore developed methods and vectors to produce homogeneous hESC lines with fluorescent and drug-selectable markers that permit isolation of pure populations of labeled stem cells and hESC-derived cardiomyocytes.

Prior strategies to increase the yield of cardiomyocytes from hESCs have included optimizing culture regimens by the addition of growth factors and other reagents to direct differentiation [7], [9], [10]. Although such advances quantitatively improved the proportion of the cells that differentiate into cardiomyocytes, in most settings the yield remains between 5–25%. Strategies for enrichment have included manual dissection of beating areas [10], [11], Percoll® density gradient sedimentation [7], [12], and fluorescence activated cell sorting (FACS) of cells based on expression of a fluorescent reporter protein from cardiomyocyte gene promoters [11], [13]. Each of these strategies has drawbacks in terms of purity (density gradient sedimentation and manual dissection), viability (FACS) and scalability (FACS and manual dissection). In theory, an effective alternative is drug resistance based selection of cardiomyocytes as successfully implemented by Field and collaborators using the Neomycin analogue G418 to purify cardiomyocytes from differentiating mouse ESC cultures [14]. Genetic selection has recently been adapted to hESCs [15], and we applied this technology with a suite of lentiviral vectors and protocols for the production of stable, homogenous and clonal lines of hESCs with Neomycin, Blasticidin and Puromycin resistance cassettes for drug selection of undifferentiated stem cells and functional hESC-derived cardiomyocytes. Additional vectors were constructed to produce hESC lines with eGFP and mCherry fluorescent reporters of mesoderm and cardiac lineages and also fluorescent Histone2B (H2B) fusion proteins that allow real-time sensing of the DNA content and recognition by automated algorithms for cell screening and tracking. We used 3 criteria (electrophysiological characterization of action potentials, force generation, and gene expression profile) to validate that the isolation procedure did not adversely affect the cardiomyocytes, indicati that the drug-isolated cardiomyocytes were physiologically normal.

## Results

### Isolation of homogeneous lines of reporter hESCs

**Figure 1:**
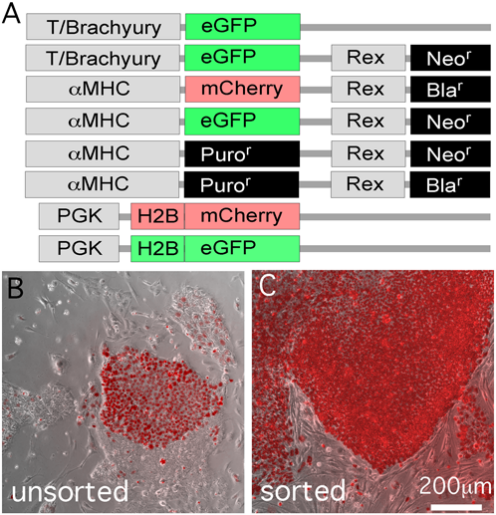
Lentiviral transduction and FACS enrichment of hESCs. (A) Lentiviral constructs used in this study. Structural details are provided in Supplemental Figures S3, S4, S5, S6, S7, S8, S9, S10 and Methods. (B,C) Merged brightfield phase contrast micrographs of mCherry-positive cells overlayed with fluorescence images illustrate the variegated expression in a colony before (b) and reduced variability after (c) FACS enrichment (see Materials and Methods). hESCs were maintained on unlabeled MEFs.

**Figure 2:**
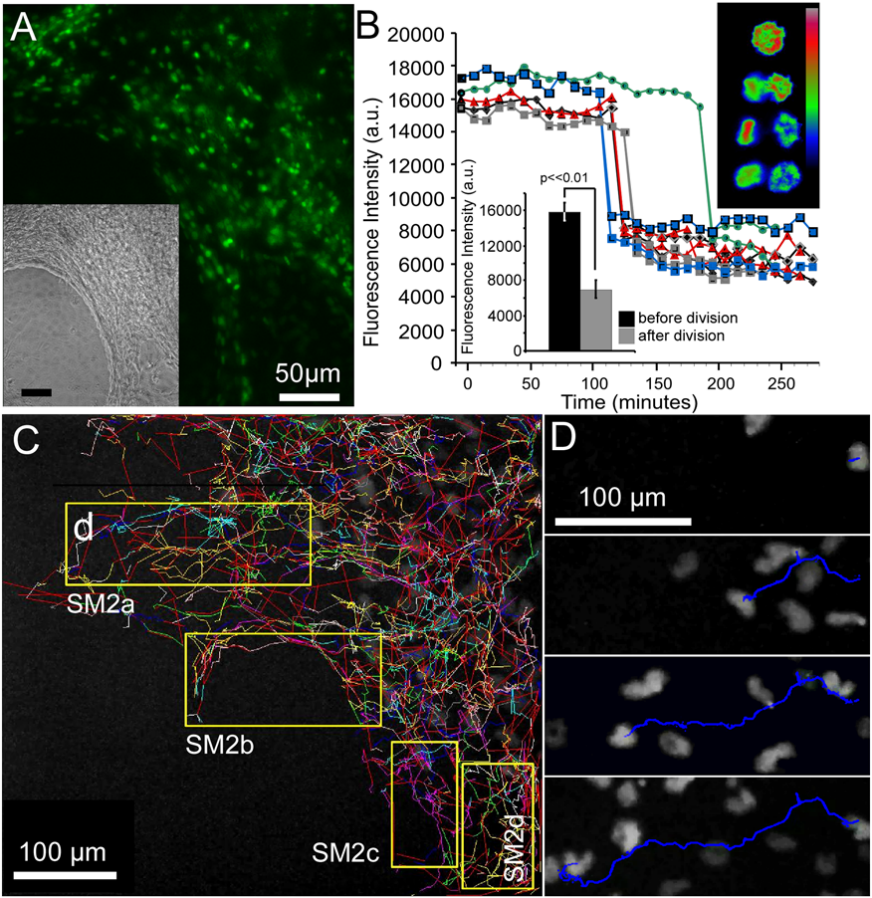
Nuclear labeling and DNA content tracking with Histone-2B fluorescent fusion protein. (A) Fluorescence and brightfield micrographs showing uniform PGK-H2BeGFP fluorescence in nuclei of cardiomyocytes derived from a FACS-enriched stable line. Spontaneous contractile activity is apparent in Supplemental Movie S1. H2B eGFP expression correlated with DAPI by flow cytometry (R^2^ = 0.733, see text). (B) Examples of tracking of PGK-H2BmCherry hESCs cells, also from a FACS-enriched stable line, undergoing cytokinesis at the border of an undifferentiated colony. Lower left inset shows fluorescence intensity of 10 images before and 10 images after cell division of the individual cells in the larger figure (n = 5). Upper right inset shows heatmap representation of H2BmCherry fluorescence intensity of a single dividing cell. (C) Tracks calculated automatically using ImageJ Plugin [17] from time-lapse images of H2BmCherry fluorescence at the border of undifferentiated ESC colony over a 2 day period (Methods). Yellow boxes indicate frames of movie clips in Supplemental Movie S2. (D) Time frames showing a single track of the H2Bmcherry centroid (blue).

Lentiviral infection of hESCs commonly results in functional transgene expression in only a fraction of the total cells, even at high multiplicity of infection, illustrated by the typically mixed colony of mCherry-labeled and unlabeled hESCs (Figure 1B) 7 days after lentiviral infection to express an H2BmCherry fusion protein from a phosphoglycerate kinase promoter (PGK, diagrammed in Figure 1A). Undifferentiated hESCs are apparent as flat colonies (Figure 1B) against the mouse embryo fibroblast (MEF) feeder cells in the co-culture (see Methods). We also found that expansion of mixed populations often reduces the percentage of expressing cells (not shown), possibly reflecting expression from non-integrated provirus, transgene silencing or a growth disadvantage of cells with the H2B fusion proteins. For example, unsorted PGK H2BmCherry or H2BeGFP hESCs progressively lose expression, beginning with as many as 80–90% positive cells and declining to fewer than 5% by the 6^th^ passage and only a few fluorescent cells after the 7^th^ passage (not shown). A further problem with heterogeneous populations is that expression level of transgene reporters tends to be varied, for example as reported for cardiomyocyte-specific expression of eGFP [11], [13], possibly reflecting variable influences of chromatin surrounding the diverse genome integration sites. The preparation of homogeneous, and ideally clonal, populations would be expected to minimize variability and therefore be desirable if not essential for many quantitative or high throughput applications. Accordingly, we found that FACS-enriched PGK-H2BmCherry- and PGK-H2BeGFP-positive hESCs could be readily expanded and yielded non-clonal cell lines that typically showed fairly uniform fluorescence intensity when examined visually (Figure 1C). The FACS-enriched hESCs could be maintained for at least 20 passages without loss of fluorescence. FACS-enriched H2B fluorescent fusion protein hESCs retained the ability to form cardiomyocytes when differentiated in embryoid body (EB) culture (Figure 2A and Supplemental Movie S1).

Nuclear localized fluorescent proteins are useful for cell tracking. Moreover, a label that shows a temporal change, such as correlation with DNA content, can be used in correction routines for during automated tracking [16], for instance to distinguish tracks of two cells that cross paths from two daughters arising from a single progenitor. To test utility of the nuclear fluorescent proteins in tracking applications, differentiating cells of the FACS-enriched hESCs were tracked automatically using a modified version of the Particle Tracking Plugin for ImageJ [17]. Fig. 2C shows the results of analysis of time-lapse image stacks of H2BmCherry fluorescence at the border of a day 4 EB acquired at 10 minute intervals over a 20 hour period using a 10× objective and MetaMorph software (Methods). Figure 2D and Supplemental Movie S2 show examples of individual tracks of H2BmCherry centroids.

Comparison of the integrated fluorescence intensity of H2BmCherry to that of DAPI in the overall population after end-point fixation and flow cytometry yielded a squared correlation coefficient (R^2^) of 0.733, indicating that H2BmCherry brightness is not as accurate a measure of DNA content as is DAPI staining. Nonetheless, we examined whether the H2B fluorescent fusion proteins could be used to report DNA content in real time by imaging PGK-H2BmCherry cells at the border of an undifferentiated hESC colony for a period of 2 days and analyzing the levels of fluorescence intensity during instances of cell division (Figure 2B). The integrated nuclear fluorescence intensity of individual H2BmCherry-positive hESCs decreased 2-fold upon cell division (Figure 2B). Thus, although cell-to-cell and temporal variation were observed in the fixed endpoint and tracking data [note that some immediate-post-division daughters exhibited larger differences in H2BmCherry brightness (blue traces) than others (grey, green and red)]; the temporal trends of single cells during S-phase and division yield cell cycle information that might be useful for error correction routines in automated tracking [16].

### Method for clonal expansion of engineered hESC lines

**Figure 3:**
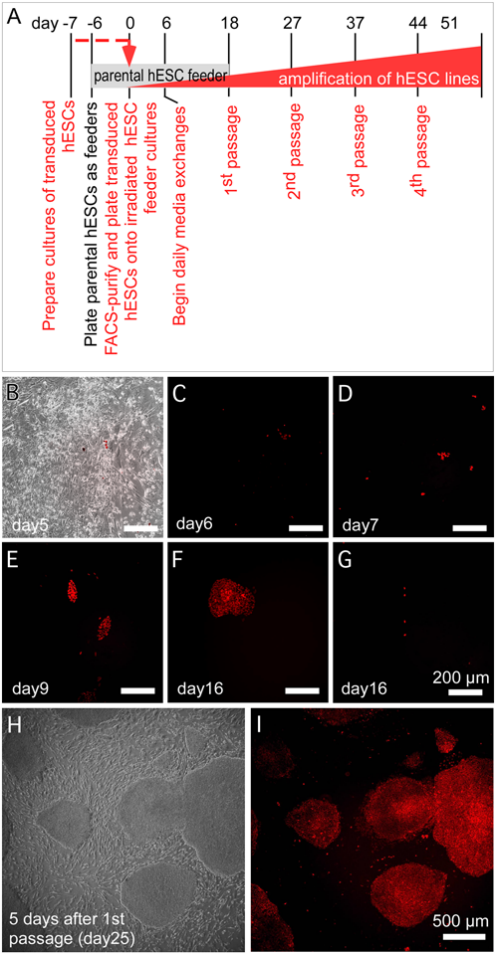
Single colony expansion of hESCs. (A) Schematic diagram of the procedure. Parental hESCs were gamma-irradiated and used as metabolically active but replication defective feeder cells. Lentivirally-transduced hESCs expressing a fluorescent reporter protein were enriched by FACS and seeded at low density on to the feeders, expanded as single colonies, and manually selected for serial passage as indicated (Methods). (B) FACS-isolated hESCs plated onto irradiated hESC feeder layer at low dilution after FACS. (C–F) Fluorescence micrographs showing examples of the sorted cells at different times after plating. Individual cells seen one day after sorting (C) grow into small colonies as imaged at days 7, 9 and 16 (D–F) (different colonies). (G) H2BmCherry-positive differentiated cells did not proliferate under the expansion conditions. (H,I) Phase contrast (H) and fluorescence (I) micrographs of clonal H2BmCherry hESCs originated from a single cell at day 7 following passage 1.

In order to create homogeneous populations of fluorescent reporter lines for quantitative applications, such as for high throughput biology or for using biosensors to measure intracellular signaling activity or subcellular constituents, we developed a protocol for clonal expansion of single cells (Figure 3A). Plating of hESCs at low seed density, such as limiting dilution for cloning, typically triggers spontaneous differentiation that leads to a loss of pluripotent cells [18]. The Rho-associated kinase inhibitor, Y-27632, has been shown to alleviate the apoptosis associated with dispersed hESCs [19]. As an additional solution to this problem, we developed a straightforward method involving seeding of FACS-isolated cells onto gamma-irradiated parental hESCs as metabolically active but replication-defective feeders (Methods). Monitoring the colonies permitted those arising from single hESCs to be chosen for serial passage and expansion. Individual clonal colonies showed reporter fluorescence during growth (Figures 3B–F). Non-stem cells did not proliferate under these conditions (Figure 3G). Recovery of colonies was efficient: approximately 1 colony could be isolated from approximately 13 cells deposited (clonal efficiency of 7.7%). In a typical experiment, of 629 total colonies obtained prior to the first passage, we monitored 24 that were clonally derived from single cells and, of these, 12 were frozen as before passage while 4 were propagated as distinct lines. Figures 3H,I illustrate the uniform H2BmCherry fluorescence of one of these colonies after the 1^st^ passage. The clonal PGK-H2BmCherry hESC lines have been maintained for over 20 passages with no discernible change in intensity level or loss of homogeneity.

**Figure 4:**
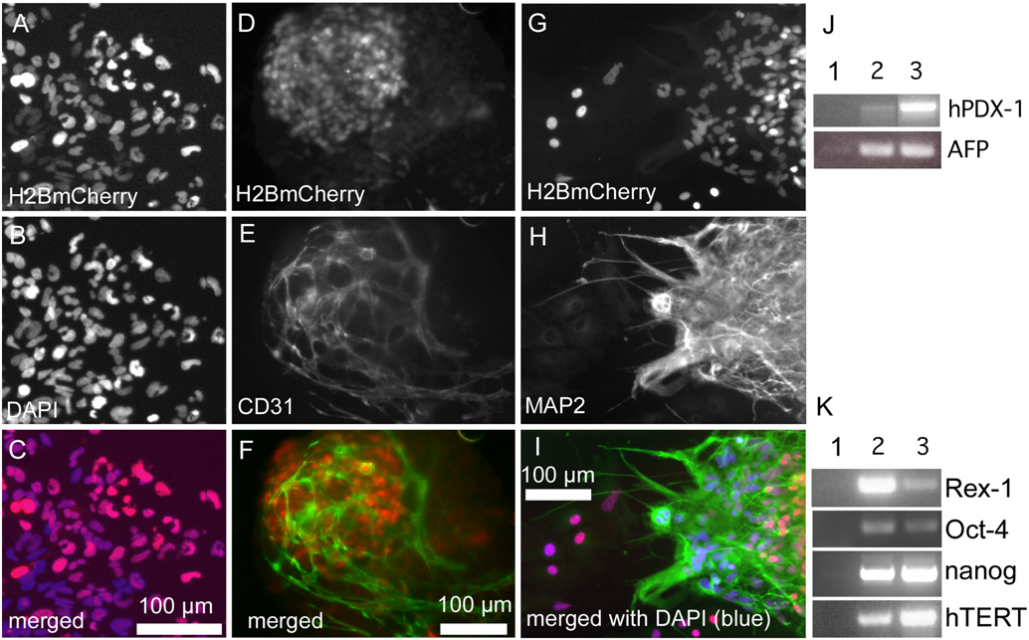
Pluripotency of clonal hESC reporter lines. (A–C) Outgrowths of differentiated cells from EBs derived from a clonal PGK-H2BmCherry hESC line showed coincident H2BmCherry (A) and DAPI (B) immunostaining visible in the merged fluorescent image (C). (D–I) Clonal PGK-H2BmCherry hESC-derived EBs developed endothelial (D–F) and neuronal (G–I) lineages. (J) Expression of endodermal genes alpha fetoprotein (AFP) and hPDX-1 by day 20 EBs of clonal PGK-H2BmCherry hESCs (lane 2) compared to undifferentiated cells (lane 1) and isolated human pancreatic islet tissue as positive control for hPDX-1 (lane 3) and day 20 parental H9 EBs as control for AFP (lane 3). (K) Pluripotency markers expressed by clonal PGK-H2BmCherry hESCs (lane 2) compared to no reverse transcriptase control (lane 1) and undifferentiated parental H9 cells (lane 3).

Engineered clonal hESCs retain pluripotency, as demonstrated by expression of hTERT, nanog, Rex-1 and Oct4 (Figure 4K) and the ability to differentiate into endothelial (Figures 4D–F), neuronal (Figures 4G–I), cardiac (see below) and endodermal (Figure 4J) lineages. Nuclear-localized H2BmCherry fluorescence is visible in each cell (compare Figures 4A,G to B,H). Taken together, these protocols and lentiviral vectors provided uniformly labeled hESCs for quantitative analyses.

### T/Brachyury promoter eGFP reporter vectors

**Figure 5:**
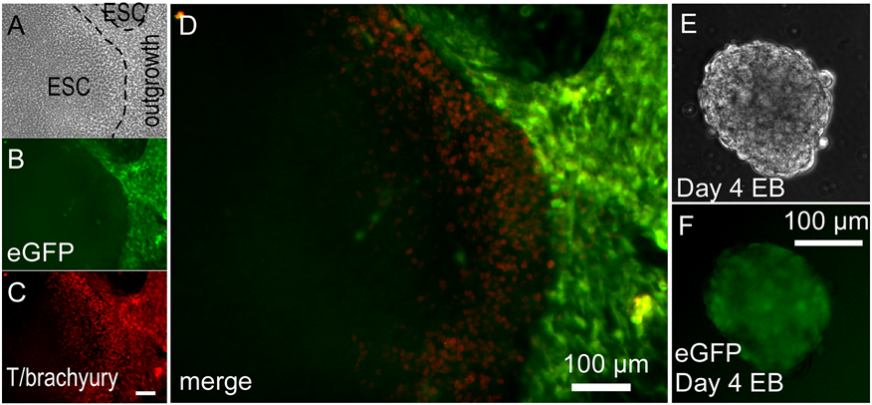
T/Brachyury eGFP hESC line. (A–D) Phase contrast (A), eGFP reporter fluorescence (B) anti-T/Brachyury immunostaining fluorescence (C) and merged fluorescence (D) micrographs of T/Brachyury-eGFP hESCs showing overlapping expression at the edge of hESCs colonies and in surrounding mesenchymal cell outgrowths. (E,F) Phase contrast (E) and eGFP reporter fluorescence (F) of day 4 differentiating EBs.

To visualize mesodermal outgrowths in hESC cultures, we used a portion of the T/Brachyury promoter [20] that contains elements shown to direct directs transcription in primitive streak-stage mesendoderm, including cardiomyocyte precursors, in transgenic mouse embryos [21]. Although absent in undifferentiated hESCs, eGFP fluorescence directed from the T/brachyury promoter vector (Figure 1A) was detectable in differentiating mesenchymal outgrowths surrounding hESC colonies (Figures 5A–D) permitting enrichment and isolation of positive colonies after infection by the clonal isolation method described in Figure 4, or by using a drug resistance procedure described in the following section. Spatially concordant immunostaining of endogenous T/brachyury protein demonstrated fidelity of the construct, although cells at the border of hESC colonies typically exhibited more endogenous T/brachyury protein than eGFP fluorescence whereas outgrowths showed intense eGFP, perhaps reflecting lagging kinetics of eGFP fluorescent detection relative to endogenous protein accumulation since this −650 to −1 region lacks sequences that are important for high level primitive streak and also node and notochord expression in transgenic mice [21], [22]. Under EB differentiation conditions, eGFP fluorescence peaked during days 3–5, also lagging behind endogenous T/brachyury expression by about a day (not shown), diminishing thereafter as expected (day 4 shown in Figure 5F). Separate studies confirmed Wnt responsiveness of this promoter region (not shown), as noted previously [20], [23].

### Drug resistance cassettes for selection of stem cells and differentiated lineages

Drug selection cassettes were developed to enable co-selection of stable hESC lines with integrated promoter-reporter constructs that are not expressed in stem cells but only in differentiated lineages. Neomycin (Neo^r^) and Blasticidin (Bla^r^) resistance genes were placed under control of the Rex-1 promoter (Figure 1A). Originally characterized in mouse embryonal carcinoma cells, Rex-1 or expression from its minimal promoter is a marker of pluripotency [24]. Rex-Neo^r^ or Rex-Bla^r^ cassettes enable co-selection of stable hESC lines with integrated promoter-reporter constructs that are not expressed in stem cells but in differentiated lineages. The Rex-Neo^r^ and Rex-Bla^r^ selection cassettes were incorporated into vectors with the cardiac-specific αMHC promoter (see Methods) directing fluorescent protein expression (e.g. αMHC-eGFP_Rex-Neo^r^ and αMHC-mCherry_Rex-Bla^r^; Figure 1A). G418 or Blasticidin selection (see Methods) of infected hESCs created non-clonal stable lines that yield fluorescently labeled cardiomyocytes upon differentiation. Supplemental Movie S3 shows an example of αMHC-mCherry expression throughout a field of spontaneously contracting cardiomyocytes. Supplemental Figure S1 shows αMHC-mCherry expression coincident with immunodetection of endogenous myosin in EBs derived from Bla^r^-selected hESCs, indicating that drug co-selection efficiently enriched functionally transduced hESCs.

**Figure 6:**
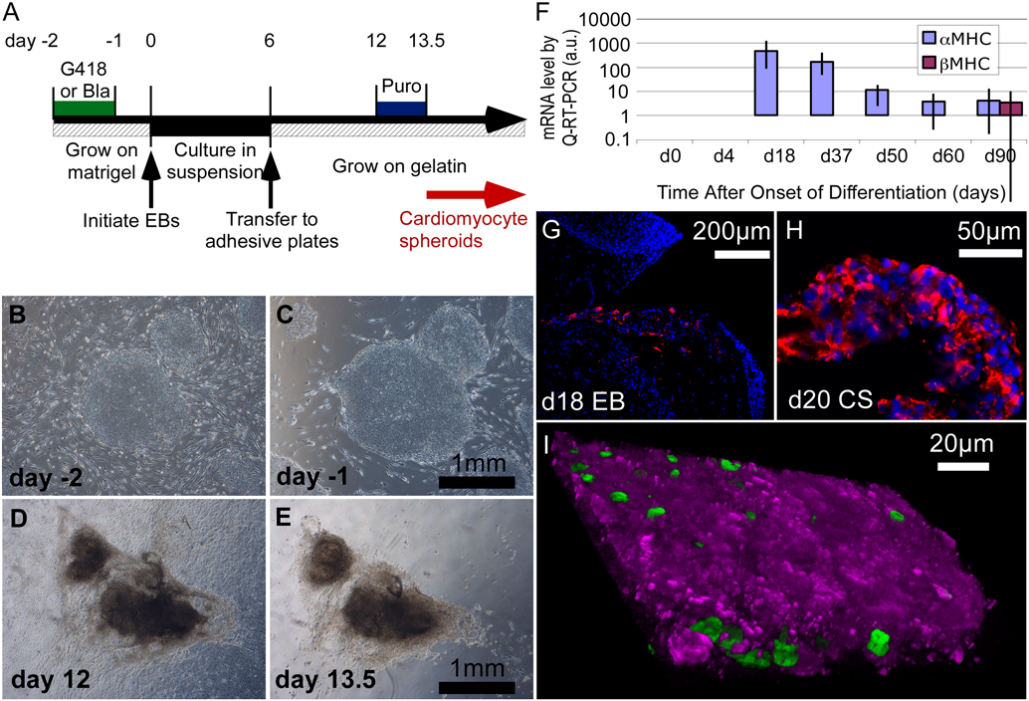
Drug resistance selection of hESC-derived cardiomyocytes. (A) Schematic diagram of the drug selection regimen for isolation of cardiomyocytes (see Methods). Puromycin selection is typically done for a 1.5–2 day window between day 12 and 120 days (latest attempted) after EB formation. (B,C) Phase contrast micrographs of hESC colonies at day −2 (b) and again at day −1, after 24 hours exposure to G418 (C). (D,E) Beating cardiomyocyte clusters at day 12 (D) and again following Puromycin treatment at day 13.5 (E). Dark color is due to opacity of the tissue. Supplemental Movie S4 shows brightfield and fluorescence images of CSs at day 16. (F) Expression of αMHC and βMHC mRNA during differentiation in EBs (day 0–4) and in Puro^r^ CSs (day 18–90), by quantitative RT-PCR (error bars represent standard deviation) showing that αMHC transcripts are evident as cardiomyocytes emerge and persist for at least 90 days, consistent with efficacy of Puro^r^ selection when performed from day 12 and up to 150 days, the latest attempted (see text). β-MHC mRNA, in contrast, was first detected at day 90. (G,H) Histological sections of a day 18 EB (G) and day 20 CS (H) showing immunostaining of Troponin-I (red) and DAPI (blue). Cardiomyocytes were enriched in CSs, comprising 96.0%±8.6, as quantified from multiple sections of 10 independent biological replicates. (I) 3D confocal reconstruction of a 16 µm section through a CS isolated from a day 100 EB by the Neo^r^, Puro^r^ selection protocol from a three-vector hESC line (αMHC-Puro^r^_Rex-1-Neo^r^, αMHC-mCherry_Rex-Bla^r^, and PGK-H2BeGFP) showing cytoplasmic mCherry (magenta) and nuclear H2BeGFP (green). Numerous nuclei are not apparent since they are inside the reconstructed tissue.

To create hESC lines for the efficient purification of cardiomyocytes, a dual selectable marker cassette lentivirus was constructed containing either the Rex-Neo^r^ or Rex-Bla^r^ cassettes together with the αMHC promoter directing transcription of the Puromycin resistance gene (αMHC-Puro^r^_Rex-Neo^r^ and αMHC-Puro^r^_Rex-Bla^r^; Figure 1A and Methods). Although βMHC is the dominant MHC in adult human heart, we chose αMHC because the onset of endogenous (Figure 6F) and transgene (Supplemental Figure S1) expression is coincident with appearance of cardiomyocytes as early as day 10, preceding expression of βMHC (detectable at day 90), and persists during subsequent culture of EBs. The Rex-Neo^r^ and Rex-Bla^r^ selection cassettes were used to create stable αMHC-Puro^r^ hESC lines that have been maintained for over 25 passages without any detectable decrease in efficacy of either selection cassette.

The dual drug selection protocol for isolation of cardiomyocytes from hESCs is diagrammed in Figure 6A and described in Methods. Two days before initiation of EB formation, cultures were treated with G418 (or Blasticidin) to remove residual MEFs and any spontaneously differentiated hESC-derived cells (Figures 6B,C). EBs were prepared for cardiogenic differentiation and subsequent selection of cardiomyocytes by Puromycin, which removed non-cardiomyocytes within 36 hours (Figures 6D,E). Puro^r^ selection could be done at any point between day 12 of culture and for up to at least 120 days, consistent with duration of αMHC expression (Figure 6F). After Puro^r^ selection, cardiomyocytes can be maintained as spheroidal clusters (cardiomyocyte spheroids, CSs) attached to the substratum (Supplemental Movie S4). CSs isolated at day 20 as were analyzed for purity by quantifying the incidence of cardiac Troponin-I (TN-I) immunostain-positive cells in histological sections. Fewer than 1% TN-I^+^ were detected in EBs (Figure 6G) whereas TN-I^+^ cells were enrichmed to 96.0%±8.6 (standard deviation; n = 10 independent biological replicates) after Puro^r^ selection (Figure 6H and [25]), consistent with previous reports of high purity of drug selected cardiomyocytes [14], [15]. Interestingly, free floating CSs eventually died while those that were loosely attached remained healthy for at least 120 days (the longest duration studied) with media exchanges every 2 days. Residual fibroblasts commonly grew out from CSs upon long-term culture (greater than 30 days) in serum-containing medium but could be removed by re-treatment with Puromycin. Puromycin treatment has been done up to 60 days following initiation of EB differentiation without loss of efficiency.

The incorporation of Neo^r^ and Bla^r^ cassettes into different vectors (Figure 1A) allowed drug selection for combinations of transgenes that, like the αMHC promoter constructs, are inactive in the stem cell state. We took advantage of this system by engineering a single hESC line with three vectors for 1) Puro^r^ selection of cardiomyocytes plus 2) visualization of cardiomyocytes with fluorescent mCherry protein and 3) visualization of all nuclei with eGFP (αMHC-Puro^r^_Rex-1-Neo^r^, αMHC-mCherry_Rex-Bla^r^, and PGK-H2BeGFP vectors). Figure 6I shows the cytoplasmic mCherry and nuclear H2BeGFP expression typical of Neo^r^, Puro^r^-selected CSs derived from this cell line. Such cell lines will permit the selection of pure populations of labeled cardiomyocytes for cell mixing, tracking and in vivo engraftment studies.

### Gene array profile of Neo^r^, Puro^r^-selected hESC-derived cardiomyocytes

**Figure 7:**
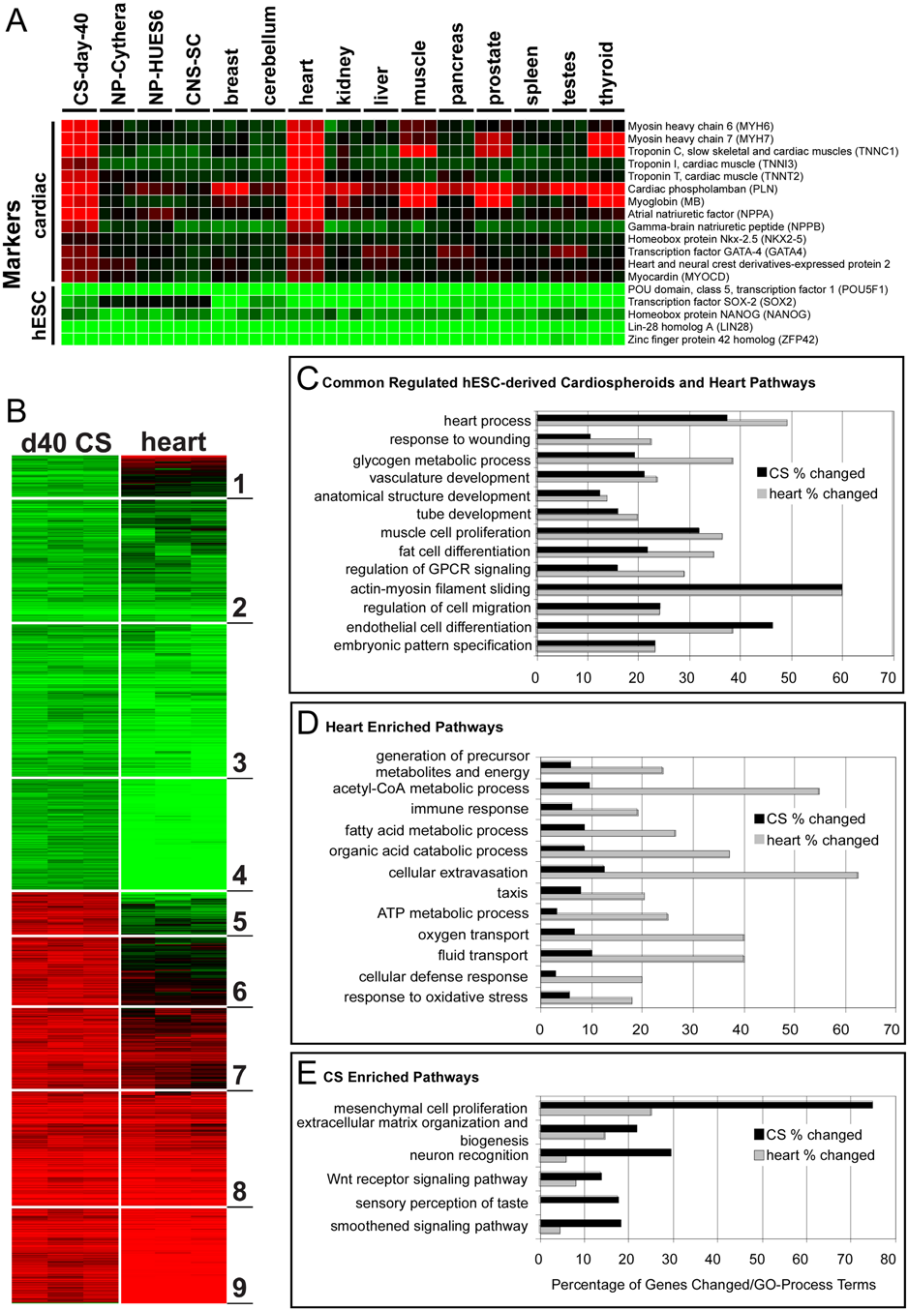
Comparison of day 40 cardiomyocyte spheroids and adult heart gene expression profiles. (A) Human Affymetrix exon microarray data for Neo^r^ undifferentiated hESCs and Puro^r^ CSs (isolated as in Figure 6a and analyzed at day 40 of culture) were combined with a previously described neural differentiation dataset (Cythera, HUES6 and fetal hCNS stem cells) in addition to adult tissue samples using the RMA algorithm (ExpressionConsole 1.0). A non-clustered heatmap of well-characterized cardiac and embryonic stem cell pluripotency genes are displayed for all combined differentiation and adult tissues, relative to their respective undifferentiated hESC controls (adult tissue compared to Neo^r^ hESCs from this study). (B) Individual array fold changes for day 40 CS and adult heart, relative to Neo^r^ undifferentiated hESCs were clustered using the HOPACH algorithm (Bioconductor), for 3030 genes up or down-regulated in the day 40 CS comparison. Genes clustered into one of nine top-level clusters (indicated), where red and green indicate gene up- and down-regulation, respectively, relative to undifferentiated hESCs. (C–E) Pathway over-representation analysis with the tool GO-Elite of the 1466 and 2502 upregulated genes in day 40 CS and adult heart samples, respectively, relative to undifferentiated hESCs as a function of the percentage of the genes changed per GO term. Cellular cardiac differentiation and complex tissue developmental pathways were enriched in both samples (C). Genes disproportionately enriched in adult heart aligned with in vivo tissue processes typical of whole heart and endothelial function (D). Genes disproportionately enriched in day 40 CS samples (E) included stem cell and early developmental processes.

A major advantage of the CS selection protocol is that it permits developmental, genetic and physiological studies of cardiomyocytes without the potentially confounding influence of other cells present in normally heterogeneous EBs. To demonstrate this approach and begin to validate that the selected cardiomyocytes retained normal function, we compared human Affymetrix exon array data from the day 40 CSs to undifferentiated hESCs, which had been treated with G418 to ensure that they were free of differentiated cells. 3030 genes were up- or down-regulated in the day 40 CSs relative to the hESCs. Further comparison of these day 40 CS profiles to adult heart arrays identified 6 out of 9 gene clusters with analogous expression profiles (Figure 7B). A subset of well-characterized cardiac and embryonic stem cell pluripotency genes are displayed in a non-clustered heatmap along with previously described neural differentiation datasets [26] and a panel of adult tissue samples (http://www.affymetrix.com/support/technical/sample_data/exon_array_data.affx) (Figure 7A). Adult heart and day 40-CS showed concordant up-regulation of all cardiac developmental and adult heart markers examined. To determine the common and distinct pathways to which these genes aligned, we performed pathway over-representation analysis with the tool GO-Elite (Figures 7C–E). Cellular cardiac differentiation pathways, as well as more complex in vivo tissue developmental pathways, were enriched among both CS and adult heart upregulated genes (Figure 7C). The adult heart had a greater proportion of up-regulated genes per corresponding gene ontology (GO) term, but with equivalent z-scores and p-values, as expected since adult heart had a larger number of upregulated genes. While the large majority of pathways were regulated in common, pathways reflecting in vivo tissue processes including cellular metabolism, immune response, taxis and fluid transport were enriched in adult heart over day 40 CS samples (Figure 7D), as expected since they correspond to whole heart and endothelial cell function missing in the CSs. For pathways in which CS genes were up-regulated and heart genes were not, neural, stem cell and early developmental processes were the most enriched (Figure 7E). Similarity of hESC-derived cardiomyocytes to adult cardiomyocytes was also described by Synnergren et al. [27], who analyzed manually dissected beating areas that are enriched for cardiomyocytes but also would be expected to include a large percentage of non-cardiomyocytes. Thus, this is the first analysis of pure hESC-derived cardiomyocyte gene expression data and the gene data can be downloaded (Supplemental Data).

### Neo^r^, Puro^r^-selected hESC-derived cardiomyocytes exhibit action potentials consistent with early embryonic fetal cardiomyocytes

**Figure 8:**
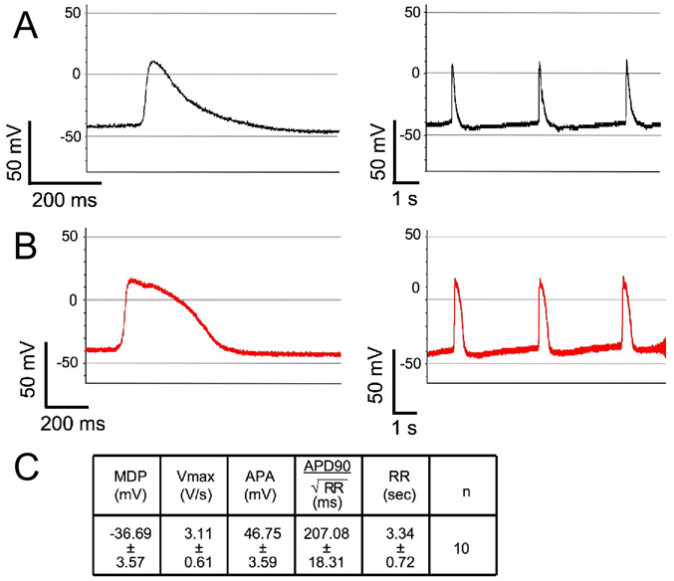
Electrophysiological properties of Neo^r^, Puro^r^-selected, hESC-derived cardiomyocyte spheroids at day 20 of differentiation. (A) The dominant electrophysiological phenotypes of action potentials (APs) of cardiomyocytes recorded from day 20 CSs. (B) Some cardiomyocytes displayed more hyperpolarized MDP and faster Vmax. Left panels (a and b) show expanded time scale and right panels show 3 APs. (C) The summary table of electrophysiological parameters of action potentials.

We next verified that the drug selection protocol does not adversely affect the electrophysiological phenotypes of the cardiomyocytes. Electrophysiological phenotypes of cardiomyocytes in the Neo^r^, Puro^r^-selected CSs at day 20 of differentiation (8 days after Puromycin treatment of day 12 EBs) were obtained by intra-cellular recording techniques (see Methods). As shown in Figure 8A, the majority of selected cardiomyocytes displayed action potentials (APs) with relatively depolarized maximal diastolic potentials (MDPs, >−45 mV) and slow maximal rate of AP depolarization (Vmax, <5 V/s). Figure 8B shows that 10–20% of cardiomyocytes, however, possess MDP<−45 mV and faster Vmax (>5 V/s). The electrophysiological parameters of these APs are summarized in Figure 8C. Overall, the electrophysiological properties of the CS cardiomyocytes were similar to those of human fetal cardiomyocytes [28].

### Force generation of isolated hESC-derived cardiomyocytes

**Figure 9:**
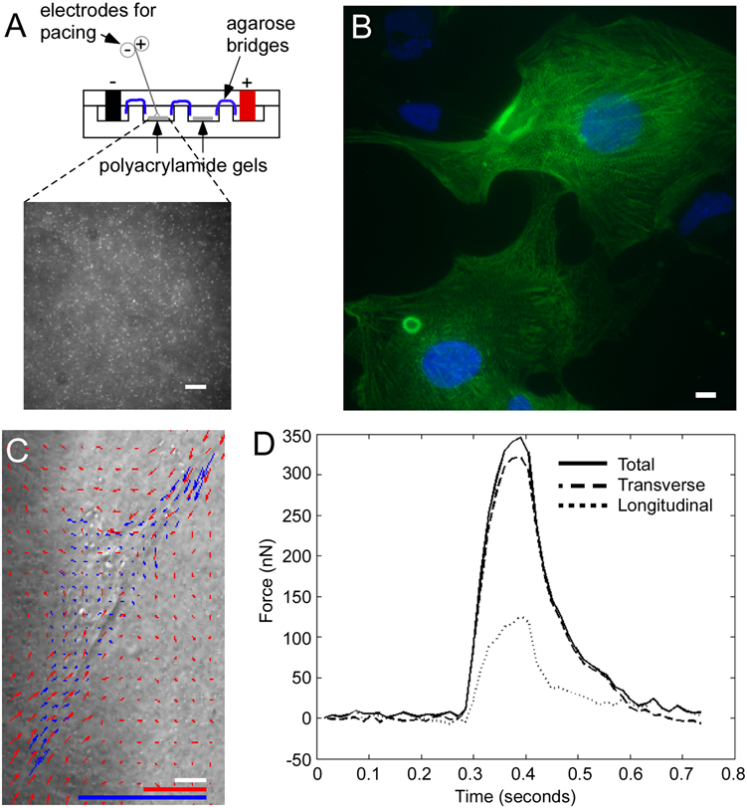
Force generation of individual day 50 Neo^r^, Puro^r^-selected cardiomyocytes. CSs were isolated at day 12–13.5 and cultured until day 48 when they were dispersed and deposited onto gelatin-functionalized surfaces of polyacrylamide cast with fluorescent beads and analyzed for force generation at day 50. (A) Diagram of the apparatus containing the polyacrylamide. Platinum electrodes were used to electrically pace the cardiomyocytes at 0.5 Hz with 0.8 ms pulses of 50 volts. Inset shows micrograph of fluorescent beads. Scale bar represents 10 µm. (B) α-actinin immunostaining of hESC-derived cardiomyocytes on the functionalized surface shows characteristic striations. Scale bar represents 10 µm. (C) Bead displacements near individual cardiomyocytes (Supplemental Figures S2A,B and Movie S4) were tracked using a cross-correlation-based optical flow algorithm in order to map deformations (red arrows) or stresses (blue arrows) across the face of the gel corresponding to individual cardiomyocytes [29]. Red arrows mark local bead displacement length is as per red scale bar (1 µm), which is expanded 20× relative to that of the image (white bar represents 10 µm) to permit visualization. Blue arrows indicate force magnitude as per blue scale bar [1 nN/µm2 (equivalent to 1 kPa)]. (D) Sample plot of total, transverse and longitudinal force versus time for a cardiomyocyte as in (C).

As a third validation of the dual drug selection protocol, contractile forces in individual, selected cardiomyocytes were measured using a method of dynamic traction force microscopy as described [see Methods and 29]. CSs were dispersed and individual cardiomyocytes plated onto polyacrylamide gels (Figure 9A) with an elastic modulus of 4 kPa and surfaces functionalized with chemically cross-linked, gelatin (Methods). Individual cells were paced (0.5 Hz with 0.8 ms pulses at 50 V) and images of fluorescent bead displacements caused by the cardiomyocytes at the gel surface were acquired every 15 ms (Supplemental Movie S5). Bead displacements were tracked using a cross-correlation-based optical flow algorithm (Methods) and mapped in order to determine localized stresses, or traction forces. Figure 9B shows a-actinin immunostaining revealing the characteristic striations of the plated cardiomyocytes and Figure 9C shows an example of bead displacements (red arrows) correlated with traction forces (blue arrows). Average traction force magnitude in hESC-derived cardiomyocytes was 220±70 Pa and was similar to that of neonatal rat ventricular cardiomyocytes (NRVCs) of 340±70 Pa. The traction forces were integrated over the projected cell area in order to calculate axial and total force. A sample plot of axial force as a function of time is shown in Figure 9D. Selected hESC-derived cardiomyocytes (N = 11) contracted with an average axial force of 139±29 nN and a total force of 144±33 nN. These values did not change appreciably with time in culture after isolation (up to 90 days, not shown). For comparison, NRVCs contracted with an average axial force of 202±47 nN and a total force of 222±54 nN on the same gels using the same protocol (Supplemental Figure S2C). Cell spread area between these cells is similar and the differences in total force are primarily due to differences in generated traction. NRVCs had a higher aspect ratio, calculated as the length of the long axis divided by the length of the short axis, than the hESC-derived myocytes, of 4.2+/−0.2 versus 3.0+−0.3. However, the ratio of the axial to total force was 0.93+/−0.02 in the hESC-derived cells and 0.93+/−0.02 in NRVCs, indicating that variations in morphological alignment did not impact the polarization of force generation. Average velocity of contractions was measured as 6.9±2.1 µm/s for hESC-derived cardiomyocytes and 9.7±1.5 µm/s for NRVCs. Thus, drug-selected hESC-derived cardiomyocytes generated contractile force comparable to that of NRVCs providing further evidence of normal behavior after selection from the engineered hESCs.

## Discussion

We describe single cell cloning and drug selection procedures methods and a suite of lentiviral vectors to engineer hESC lines for visualization, tracking and purification of pluripotent stem cells and their differentiated cardiomyocyte derivatives. These methods overcome variegation and downregulation of fluorescent reporters and other markers observed without enrichment (Figure 1 and reported previously [11], [13]). The FACS and cloning protocols yielded homogeneous levels of fluorescent reporter protein expression sufficient for automated tracking and quantitative analyses, such as biosensors of DNA content (Figure 2B), and the protocols and vectors could be broadly applied to biosensors of subcellular structures or signal transduction pathways (such RhoA fluorescence resonance energy transfer biosensors [30]) as well as improving the identification of hESC derivatives after engraftment in animal models of disease or regeneration.

Although the experiments shown were performed using H9 cells (WiCell WA09), similar outcomes were obtained with the PGK-H2B fluorescent protein vectors, Rex- and αMHC-driven fluorescent and selectable markers using HUES13 (Harvard) and human induced pluripotent stem cells (hIPSCs) derived in our laboratory.

Dual cassette lentiviral vectors were developed to enable drug selection of pluripotent stem cells with G418 or Blasticidin and cardiomyocytes with Puromycin. The two-stage selection procedure (Figure 6) first eliminated feeder and spontaneously differentiating cells, which in our hands compromise cardiomyocyte differentiation in EBs (not shown). Puromycin selection yielded greater than 96% pure cardiomyocytes at or after day 12. We exploited the purification procedure to profile hESC-derived cardiomyocytes gene expression with the finding that their profile strongly resembled that of the adult human heart. Additionally, the selected cardiomyocytes exhibited action potentials that resemble human fetal cardiomyocytes. Additional electrophysiological analysis of the early hESC-derived cardiomyocytes (Puromycin treatment at up to 20 days) reveals that the typically immature calcium handling apparatus and the proportion of cells with immature (versus ventricular-like) action potentials do not change with continued culture; however, we found that culture within the context of the EB promotes maturation of these electrophysiological parameters [25], implicating critical signals from non-cardiomyocytes.

The hESC-derived cardiomyocytes generated contractile forces characteristic of fetal cardiomyocytes, illustrating the utility of the approach for developmental and physiological studies and providing further validation that the cardiomyocytes produced by drug selection from the engineered hESCs were normal by multiple physiological criteria. It should be noted that these measurements were performed on gels with mechanical properties (4 kPa) much softer than the adult myocardium (10–20 kPa) in order to improve the resolution of the dynamic traction force microscopy, which increases as the shortening length increases, and also to approximate conditions for fetal myocardium, which has less collagen content than adult heart. Reduced stiffness is expected to result in lower force because increased shortening decreases force generation. It is to be expected that more mature cells would generate much greater contractile force. For comparison, we found that adult cardiomyocytes from rabbits at slack length contraction, with sarcomere lengths near 1.8 µm, generate forces ranging from 500 nN to 2700 nN depending on the stimulation frequency[31]. Active stresses in these cells are again an order of magnitude greater than the hESC-derived cardiomyocytes, ranging from 3000 to 5000 Pa. In addition, the two-dimensional geometry of this cell culture could further decrease the force generated compared to a more physiological three-dimensional environment.

Cell tracking using the PGK-H2BmCherry- and PGK-H2BeGFP-containing vectors will enable stem cells and their differentiated derivatives, regardless of lineage, to be followed in cell mixing and in vivo engraftment studies. The heritable and ubiquitously expressed reporters can be used to quantify regeneration of functional cardiomyocytes as well as persistence of non-cardiomyocyte derivatives of the graft, such as fibroblasts or de-differentiated cells as well as residual stem cells with tumorigenic potential. Moreover, application of the H2B fluorescent fusion proteins as sensors of DNA content in a 2-D tracking study (Figure 2B) showed feasibility of using the reporter to evaluate parameters such as cell migration and differentiation that also should be applicable to studies of tissue architecture in 3-D cultures, as recently reported [32].

In conclusion, we have described a toolbox of lentiviral vectors and protocols for cell line creation and isolation of pure cardiomyocytes that provide the means for a wide range of studies aimed at improving cardiomyocyte differentiation from stem cells and their functional incorporation into damaged myocardium.

## Methods

### hESCs and culture conditions

Low passage hESCs (H9, WiCell) were used. Similar results were obtained using HUES13 (Harvard) and hIPSCs derived in our laboratory. Undifferentiated hESCs were cultured as described [33] with slight modification. Briefly, cells were cultured in Knockout Dulbecco's modified Eagle's medium (KODMEM, Invitrogen, 10829-018) supplemented with 1 mM L-glutamine with 20% Knockout Serum Replacement medium (KOSR, Invitrogen), 1 mM sodium pyruvate, 0.1 mM nonessential amino acids (NEAA, Invitrogen), 50 U/ml penicillin, 50 µg/ml streptomycin (Invitrogen), 0.1 mM beta-mercaptoethanol (Invitrogen) and 8 ng/ml basic fibroblast growth factor (bFGF, Sigma catalogue F0291-25UG). hESCs were grown on Matrigel (growth factor-reduced, BD Bioscience)-coated 6-well plates (Corning, Inc. catalogue 3506) on a feeder layer of primary MEFs from E13.5 CD-1 mice isolated as described [33]. Passage 3 to 4 MEFs were gamma-irradiated with 3,000 rads (30 Grays) and plated at 10^4^ cells per cm^2^. All hESC lines were passaged following enzymatic digestion with either collagenase IV (Invitrogen, 17104-019) approximately every 7 days or Accutase (Chemicon) approximately every 10 days [34], depending on cell condition and confluency. For collagenase treatment, cells were exposed to 1 mg/ml in KODMEM, sterile filtered, at room temperature. Once the edge of colonies were about to lift from the plate, the cells were rinsed twice with DPBS (Ca^2+^- and Mg^2+^-free), culture medium was added and cells were mechanically dispersed into 100–500-cell clusters by trituration using a 5 ml pipette and re-plated. For Accutase treatment, cells were washed twice with DPBS and then subsequently washed with a small amount of Accutase (1× concentration, Innovative Cell Technologies) and then exposed to Accutase at room temperature. After a few minutes, when MEFs and hESC-derived fibroblasts began to lift from the plate, accutase was removed and hESCs were washed twice with DPBS (Ca^2+^- and Mg^2+^-free) to remove MEFs and hESC-derived fibroblasts. A third of the volume of culture medium normally used was added and the stem cells were mechanically dispersed into 10–50-cell clusters by trituration as above. Each passage was a 1∶3 split ratio for collagenase IV-treated cells and 1∶4 to 1∶6 ratio for accutase-treated cells. Cells were routinely tested for mycoplasma (MycoAlert; Cambrex, Walkersville, MD).

### Lentivirus vector design, preparation and hESC infection

The SIN18.WPRE lentiviral vector [35] was modified by insertion of the promoter regions and the drug selectable or fluorescent proteins (Figure 1A; see Supplemental Figures S3, S4, S5, S6, S7, S8, S9, S10 for individual schematics). Vectors and schematics are available at *[insert web site of public source once available]*. The lineage-specific vectors included gene promoters for T/Brachyury [20] [−645 bp to −1 bp relative to ATG (includes 152 bp of 5′UTR)] and αMHC [36] [−5446 bp to −4 bp relative to ATG (includes non-coding exons 1,2 and UTR of exon 3)] with the stem cell selective Rex-1 (also known as zinc finger protein 42) promoter [24] (−1062 bp to −357 bp relative to ATG) to direct the selectable markers. The ubiquitously expressed human PGK promoter (−528 bp to −13 bp relative to ATG) directed expression of the H2B-fluorescent fusion proteins.

SIN18.WPRE-based lentivirus production in HEK 293T cells was as previously described [35], [37], followed by purification and concentration by ultra-centrifugation. Briefly, three plasmids (transfer vector with expression construct, the packaging plasmid pCMVΔR8.74, and the VSV-G envelope protein expression plasmid pMD.G) were mixed in a ratio of 3∶2∶1 and 293T cells were transiently transfected using calcium phosphate method and viral supernatant from the transfected plate was collected every 24 hours in serum-free Ultraculture medium (Bio-Whitttaker #12-725F) with 1 mM L-glutamine, 50 U/ml penicillin, 50 µg/ml streptomycin up to 4 days after the transfection. The pooled viral supernatant was concentrated by ultracentrifugation at 21,000 rpm for 2 hours at 4°C, passed through 0.22 or 0.4 µm filters, and aliquots were used to transfect the hESCs.

For infection, confluent hESCs, in one well of a 6-well plate, were lightly dissociated with 1 mg/ml collagenase 7 days after the last passage and rinsed twice with DPBS. The small cell clumps of approximately 100 to 200 cells were resuspended in 1 ml of culture medium and collected upon settling in 15 ml conical tube for 5 min at room temperature. 500 µl of the supernatant was exchanged with fresh 400 µl fresh media and 8 µg of polybrene. Finally 100 µl of the concentrated virus supernatant was added and mixed with the cells and incubated at 37°C for 4 to 6 hrs. The cell/virus suspension was mixed occasionally during the incubation and then plated on to one or two wells of the Matrigel-coated wells with MEF cells and cultured overnight. 1 ml of the culture media was added to the cells on the next day and the virus particles were washed out 36 hours after the infection by medium change.

### G418 and Blasticidin selection of drug resistant hESC lines

Four days after virus infection, hESCs were treated with either G418 (400 µg/ml) or Blasticidin (5 µg/ml) for 36 hours, rinsed twice with DPBS to remove drugs, and cultured for two to three days with daily medium change to permit recovery. Recovered cells were then treated with the same drug a second time and allowed to recover, as before, until colonies attained sufficient size and cell density for passage.

### FACS purification and cloning of hESCs

Figure 3A diagrams the FACS isolation and clonal expansion procedure. Plates of irradiated feeder hESCs were set up six days prior to FACS of the cells intended for clonal expansion by plating parental hESCs onto Matrigel-coated 6-well plates under regular maintenance culture conditions with MEFs as above. On the morning of the day when needed as feeders, these plates were gamma-irradiated with 3,000 rads (30 Grays), rinsed twice with DPBS, and medium exchanged with fresh H9 maintenance medium.

To generate a single-cell suspension for FACS, hESCs were dispersed with Accutase (1×) for 10 to 15 minutes at room temperature and cells were collected by centrifugation at 200 rpm for 5 minutes and the medium was exchanged to regular culturing medium and kept at room temperature until use. The dissociated hESCs (adjusted to 10^6^ cells/ml) were stained with SytoxGreen (Invitrogen) or 7-AAD (7-amino-actinomycin D, BD Bioscience) prior to sorting on a FACSVantage™. Cell debris, cell clumps, dead cells and MEFs were gated out before sorting. Dissociated hESCs were sorted in pre-warmed 100% KOSR and then diluted with pre-warmed culture media and seeded on top of the irradiated hESC feeder plates at 10,000 to 20,000 cells/well final concentration on Matrigel-coated 6-well dishes (Corning, Inc. catalogue 3506) with 20% and 40% of KOSR, respectively. Fresh medium was added occasionally but not exchanged until day 7 post-FACS and then exchanged every day thereafter. Single colonies were passaged onto irradiated MEFs in a well of a 24-well dish on day 18 after which they were expanded onto successively larger wells with each passage (Figure 3) with clonal cells reaching confluence in 6-well format approximately 50 days post-FACS.

### hESC differentiation

Figure 6A schematically illustrates the protocol for obtaining Neo^r^, Puro^r^ (or Bla^r^, Puro^r^) cardiomyocytes. Undifferentiated hESC colonies were plated onto Matrigel-coated 6-well dishes (Corning Inc. Cat No. 3506) that had been seeded with 42,000 MEF cells/well and cultured until use. Prior to initiation of EB formation, the cells were treated with G418 (400 µg/ml; 1×) or Blasticidin (5 µg/ml; 1×) for 36 hours to remove residual MEFs and hESC-derived fibroblasts. Because the MEFs were removed, it was necessary to exchange media at this point (day −2) and again the next day (day −1) with 50% MEF-conditioned media and 50% hESC culturing media. Differentiation was initiated by EB formation on day 0 by treatment with 1 mg/ml collagenaseIV followed by two rinses with DPBS to remove any residual MEFs. The collagenase IV-treated colonies were dispersed by mechanical pipette trituration into cell aggregates of 500 to 800 cells. Aggregates were collected into 15 ml plastic tubes in cardiogenic medium [KODMEM supplemented with 20% fetal bovine serium (FBS; Hyclone), 1 mM L-glutamine, 1 mM sodium pyruvate, 0.1 mM NEAA, 50 U/ml penicillin, 50 µg/ml streptomycin, and 0.1 mM beta-mercaptoethanol] and allowed to settle for 5 minutes at room temperature. The supernatant containing single cells and cell debris was carefully removed and the pellet rinsed twice with medium before being re-plated on low attachment plates (Corning, Inc. Costar 3171). The medium was exchanged on day 2 and on every second day thereafter. After 6 days in suspension, EBs were transferred onto 0.1% gelatin-coated bacterial culture dishes where they attached. Cardiomyocytes generally started to appear on day 9. Cardiomyocytes were purified from αMHC-Puro^r^ hESCs by treatment with 1.8 µg/ml Puromycin for 36 hours at day 12 to14 and washed twice with DPBS.

For neuronal differentiation, EBs were prepared as above but with aggregates of approximately 50 to 100 cells in neurogenic medium [DMEM/F12 medium (Invitrogen) supplemented with 0.1 mM NEAA, 1× N-2 supplement (Invitrogen, catalogue 17502), 1× B-27 supplement (Invitrogen, catalogue 17504), 25 µg/ml bFGF (Chemicon)] in place of cardiogenic EB medium.

For endodermal differentiation, EBs were prepared as above but with aggregates of approximately 100 to 200 cells in endodermal EB medium [DMEM/F12 medium supplemented with 20% FBS (Hyclone), 0.1 mM NEAA, 0.1 mM beta-mercaptoethanol].

### RT-PCR

Total RNA was extracted using acid-guanidium-phenol-chloroform and cDNA was synthesized using the QuantiTect Reverse Transcription kit (Qiagen) and amplified products measured by Syber Green incorporation on the LightCycler (Roche). The following primers were used: hAMHC_U5619, GAAGGGCATGAGGAAGAGTGA; hAMHC_L5901, GGTTATTCCTCGTCGTGCATC; hBMHC_U5242, AGAACACCAGCCTCATCAACC; hBMHC_L5639, CTGTCCTCCTCCGTCTGGTAG; hbACTIN_U, GAGCATCCCCCAAAGTTCACA; hbACTIN_L, GCAATGCTATCACCTCCCCTG; hPDX-1_U, CCGCAGGAACCACGATGAGA; hPDX-1_L, GCCACAAACAACGCCAATCC; hAFP_U, GTCGTTTTGTCTTCTCTTCC; hAFP_L, GCCACAAATAACAGAGGAAC. Oct-4, Nanog, Rex-1 and hTERT primers were as in [38].

### Immunohistochemistry

Cells were washed with warm PBS, fixed with ice-cold MeOH at −20°C for 7 minutes and then incubated with DPBS for 10 minutes at room temperature. Cells were blocked with 1%BSA/PBS for 1 hour and then incubated in primary antibodies for 1 hour at room temperature. After three 10-minute washes with PBS, the secondary antibody solution was incubated for a period ranging from 40 minutes to overnight at 4°C and then washed three times with PBS prior to mounting with SlowFade mounting medium with DAPI (Invitrogen). Histological sections were sectioned in OCT at 8 µm and stained as above to quantify percentage of cardiomyocytes in CSs. Cardiac Troponin-I (Alomone Labs), MAP2 (Chemicon), CD31 (eBiosciences), and appropriate AlexaFluor488 (Invitrogen), Cy3 or Cy5 (Jackson ImmunoResearch) secondary antibodies were used for immunostaining.

### Microscopy and DNA content determination

For cell tracking and DNA content determination, differentiating hESCs were plated in 2 ml of appropriate differentiation medium for two days prior to recording onto 0.17-mm thick Delta T glass-bottom culture dishes (Biotechs, Butler, PA) that had been coated with 0.1% gelatin for 1 hr at room temperature. The dishes were then sealed with parafilm and mounted on the stage of an inverted Nikon microscope equipped with electronically controlled shutters, filter wheels, and a 14-bit cooled CCD camera (Orca II, Hamamatsu Corporation) controlled by MetaMorph software (Molecular Devices, USA). Time-lapse images were acquired for up to several days at a time. H2BmCherry, H2BeGFP and DAPI integrated fluorescence intensity was calculated and cell tracks were created using MetaMorph and a modified version of Particle Tracking Plugin for ImageJ [17].

### Gene Expression Microarray Analysis

Total RNA was extracted as described [26] for biological triplicates of Rex-Neo^r^ hESCs and Rex-Neo^r^, αMHC-Puro^r^ day 40 CSs for microarray sample preparation. Total RNA with a concentration of ∼1 µg, was treated with the RiboMinus human Transcriptome Isolation kit (Invitrogen) and used as input for the GeneChip® WT cDNA Synthesis and WT Terminal Labeling kits (Affymetrix), according to manufacturers instructions by the Gladstone Institutes Genomics Core. The resulting fragmented and labeled cDNA were hybridized to individual Human Exon 1.0 ST GeneChip arrays and scanned according to manufacturers' instructions. Affymetrix CEL files from these samples were combined with CEL files for the Cythera neuronal precursor differentiation datasets (Cy-ESCs and Cy-NPs), HUES6 cell line experiment (HUES6-ESCs and HUES6-NPs) and fetal human CNS stem cells (hCNS-SCs), provided by the Gage laboratory (http://www.snl.salk.edu/~geneyeo/stuff/papers/supplementary/ES-NP) and 33 CEL files for 11 different adult human tissues obtained from the Affymetrix website (http://www.affymetrix.com/support/technical/sample_data/exon_array_data.affx).

RMA [39] expression values and detection p-values were obtained for all probesets using the Affymetrix program, ExpressionConsole (http://www.affymetrix.com/products/software/specific/expression_console_software.affx). To calculate gene expression values from the exon array data, we developed a program in python called ExpressionBuilder. Expression builder aligns probeset genomic coordinates to Ensembl genes and exons along with probeset to transcript associations from the Affymetrix probeset annotation file (HuEx-1_0-st-v2.na23.hg18.probeset.csv) to identify probesets that are most common (constitutive) to all transcripts for an Ensembl gene. Constitutive gene expression values were determined from the mean of the probeset log2 intensity values of all constitutive probesets. If no constitutive probesets are present, gene expression is calculated by the mean of all gene linked probeset intensities. To determine differential expression, fold changes and T-test p-values were calculated from the log2 expression data for differentiated cell sample arrays compared to the appropriate undifferentiated hESC baseline (Rex-Neo^r^ H9, Cythera or HUES6 lines).

Differentially expressed genes (absolute fold>2 and p<0.05) for day 40 CS samples compared to Rex-Neo^r^ hESCs were clustered along with differentially expressed genes (same criteria) in adult heart compared to Rex-Neo^r^ hESCs (no filtering) using the clustering method HOPACH (hierarchical ordered partitioning and collapsing hybrid) in R [40]. The resulting cluster data was visualized in the program TreeView [41]. Gene Ontololgy over-representation analysis and tree filtering were performed using the freely available software GO-Elite (http://www.genmapp.org/go_elite/go_elite.html).

### Downloadable Gene Expression Dataset

The hESC and tissue derived gene expression data can be downloaded at http://conklinwolf.ucsf.edu/informatics/Mercola/DATASET-all-tissues_all-hESCs_all_diff-rma-exon.zip. For 3472 Ensembl gene identifiers, mean fold change and ttest p-values are provided along with log2 expression values for all in vitro and in vivo cell/tissue derived exon arrays. This data is accompanied by gene annotations including probesets for which the values are derived, associated Affymetrix transcript clusters and HOPACH cluster data (used in Figure 7).

### Intracellular recordings with sharp electrode technique

CSs were plated on coverslips coated with 0.1% gelatin and the coverslips were mounted in a chamber on the stage of an inverted microscope (Olympus IX71) and superfused with extracellular DMEM containing 1.8 mM Ca^2+^. All experiments were conducted at 37°C and the extracellular DMEM was continuously pre-oxygenated with 95% O_2_/5% CO_2_. Sharp glass microelectrodes are fabricated with resistances of 50–200 MΩ when filled with 3 M KCl. The spontaneously beating CSs were then impaled with the microelectrodes and electrode capacitance was nullified. The intracellular recordings of APs were obtained using an AxoPatch 200B amplifier in current clamp mode and pCLAMP-10 software (Molecular Devices). Data were sampled at 10 kHz and low pass filtered at 5 kHz. The following parameters of APs with more than 10 seconds of stable baselines were measured: AP amplitude (APA), maximum diastolic potential (MDP), maximal upstroke velocity (Vmax), AP duration at 90% of the repolarization (APD90), and the cycle-length between two spontaneous APs (RR). The APD90 is corrected by heart rates with Bazett formula (APD/square root of RR).

### Single cell force measurements

Contractile forces in individual cells were measured using a method of dynamic traction force microscopy [29]. Briefly, polyacrylamide gels with an elastic modulus of 4 kPa were polymerized using 4% acrylamide (BioRad), 0.2% bisacrylamide (BioRad), 5% fluorescent beads (Molecular Probes), 0.1% ammonium persulfate (Sigma) and 0.5% N,N,N′,N′-Tetramethylethylenediamine (TEMED, Biorad). These gels were bound to cover slips coated with 3-aminopropyltrimethoxysilane (Sigma) and 0.5% glutaraldehyde (Sigma). Gels were coated with 0.5 mg/ml of rat tail type I collagen (Sigma) bound through the heterobifunctional crosslinker N-Sulfosuccinimidyl-6-[4′-azido-2′-nitrophenylamino] hexanoate (sulfo-SANPAH, Pierce). Contractile CSs were manually transferred to 0.1% gelatin-coated plates for 24 hours and then dispersed into single cells by incubating in 0.25% Trypsin-EDTA (Gibco) for 30 minutes at 37°C. Individual cardiomyocytes were resuspended in serum-containing media and plated onto collagen-coated polyacrylamide gels at a density of 20,500 cells per gel and allowed to attach to substrates overnight.

Individual cells were stimulated at 0.5 Hz with 0.8 ms pulses of 50 V using a platinum electrode. Images of fluorescent beads at the gel surface were taken every 15 ms (Supplemental Movie S5). Bead displacements were tracked using a cross-correlation-based optical flow algorithm in order to map deformations across the face of the gel [42]. These displacements, along with the gel elastic modulus and Poisson Ratio were used to calculate a map of shear stress on the gel surface based on the Boussinesq solution of deformation in an infinite elastic half space [43]. These stresses, or traction forces, were integrated over the projected cell area to calculate force, which is then graphed versus time. Force vectors were projected along the major axis of contraction in order to calculate the reported axial force.

## Supporting Information

Figure S1 Co-incident fluorescent reporter and myosin expression in EBs derived from engineered hESCs. Examples of differentiating EBs from αMHC-Puror_Rex-Neor, αMHC-mCherry_Rex-Blar, PGK-H2BeGFP hESC line created by G418 and Blasticidin selection. EBs were plated onto gelatin at day 6 of differentiation and processed for immunochemistry at day 10. Micrographs of two EBs imaged at 10× (E–H) and 20× (A–D) showing DAPI (A,E), mCherry (B,F) and MF20 immunostaining (C,G) and merged (D,H) fluorescence. mCherry and MF20 immunostaining fluorescence were coincident in Blasticidin-selected hESCs, reflecting efficacy of the co-selection strategy at removing non-functionally transduced cells.
(9.04 MB TIF)

Figure S2 Bead displacement and force generation by day 50 hESC-derived cardiomycytes. Individual hESC-derived cardiomyocytes were deposited onto gelatin functionalized surfaces of polyacrylamide gels incorporating fluorescent beads (see Methods). (A,B) Fluorescent micrographs of bead surface beneath a cardiomyocyte when relaxed (A) and contracted (B). Bead displacements were plotted as deformations or stresses, with displacement vectors as red arrows. Panels are individual frames from the stack shown as Supplemental Movie S5. White scale bars (10 µm) are for the image. Red scale bars (1 µm) represent the scale of the displacement arrows that are expanded 20-fold for visualization. (C) Example of a stress map across the face of the gel during contraction of the hESC-derived cardiomyocyte causing the displacement in (B). The scale bar represents 10 um and scale arrow represents 0.1 nN/µm2. (D) Sample plot of total force over time for hESC-derived cardiomyocyte (dashed) and control neonatal rat ventricular cardiomyocyte (solid) showing similar force generation.
(2.19 MB TIF)

Figure S3 Schematic of T/Brachyury-eGFP.
(0.42 MB TIF)

Figure S4 Schematic of T/Brachyury-eGFP_Rex-Neo.
(0.45 MB TIF)

Figure S5 Schematic of αMHC-mCherry_Rex-Blar.
(0.28 MB TIF)

Figure S6 Schematic of αMHC-eGFP_Rex-Neor.
(0.45 MB TIF)

Figure S7 Schematic of αMHC-Puror_Rex-Neor.
(0.41 MB TIF)

Figure S8 Schematic of αMHC-Puror_Rex-Blar.
(0.41 MB TIF)

Figure S9 Schematic of PGK-H2BmCherry.
(0.55 MB TIF)

Figure S10 Schematic of PGK-H2BeGFP.
(0.50 MB TIF)

Movie S1 Example of uniform PGK-H2BeGFP fluorescence in nuclei of cardiomyocytes derived from a FACS isolated hESC line. Automaticity is evident by spontaneous contractions. Inset shows phase contrast brightfield image.
(1.15 MB MOV)

Movie S2 Example of cell tracking using PGK-H2BmCherry hESCs. Time-lapse image stacks of PGK-H2BmCherry ESCs over 20 hours were acquired with a 10× objective (NA 0.5) at 10 minute intervals using MetaMorph. Centroids of H2BmCherry fluorescence were tracked automatically using a modified version of the Particle Tracking Plugin [17] for ImageJ (Methods). The 4 concatenated clips correspond to single tracks within the yellow boxes in Figure 2C.
(1.52 MB MOV)

Movie S3 Example of cardiomyocytes labeled with αMHC-mCherry. The hESC line was engineered with αMHC-Puror_Rex-Neor and αMHC-mCherry_Rex-Blar vectors (Figure 1A) by G418 and Blasticidin selection (Methods). Phase contrast and fluorescence images show mCherry+ cardiomyocytes within the EB plated on gelatin-coated cell culture plastic without Puromycin treatment.
(2.45 MB MOV)

Movie S4 Example of cardiomyocyte spheroid uniformly labeled with αMHC-eGFP Example of a dual drug selected CS from a hESC line engineered with αMHC-Puror_Rex-Blar and αMHC-eGFP_Rex-Neor vectors (Methods). Phase contrast and eGFP fluorescence image stacks were acquired 3 days following Puromycin drug selection (corresponding to day 16 after EB formation, see Methods).
(1.63 MB MOV)

Movie S5 Bead displacements used to calculate axial force of individual cardiomyocytes. Bead displacements in gels were tracked using a cross-correlation-based optical flow algorithm in order to map deformations (Supplemental Figure S1B) and stresses (Supplemental Figure S1C) across the face of the gel corresponding to individual cardiomyocytes [42].
(1.36 MB MOV)
