# Supplementary figures and images for: Lentiviral Vectors and Protocols for Creation of Stable hESC Lines for Fluorescent Tracking and Drug Resistance Selection of Cardiomyocytes
Source: PLoS One. 2009 Apr 8;4(4):e5046. doi: 10.1371/journal.pone.0005046 (PMC2662416; doi:10.1371/journal.pone.0005046)

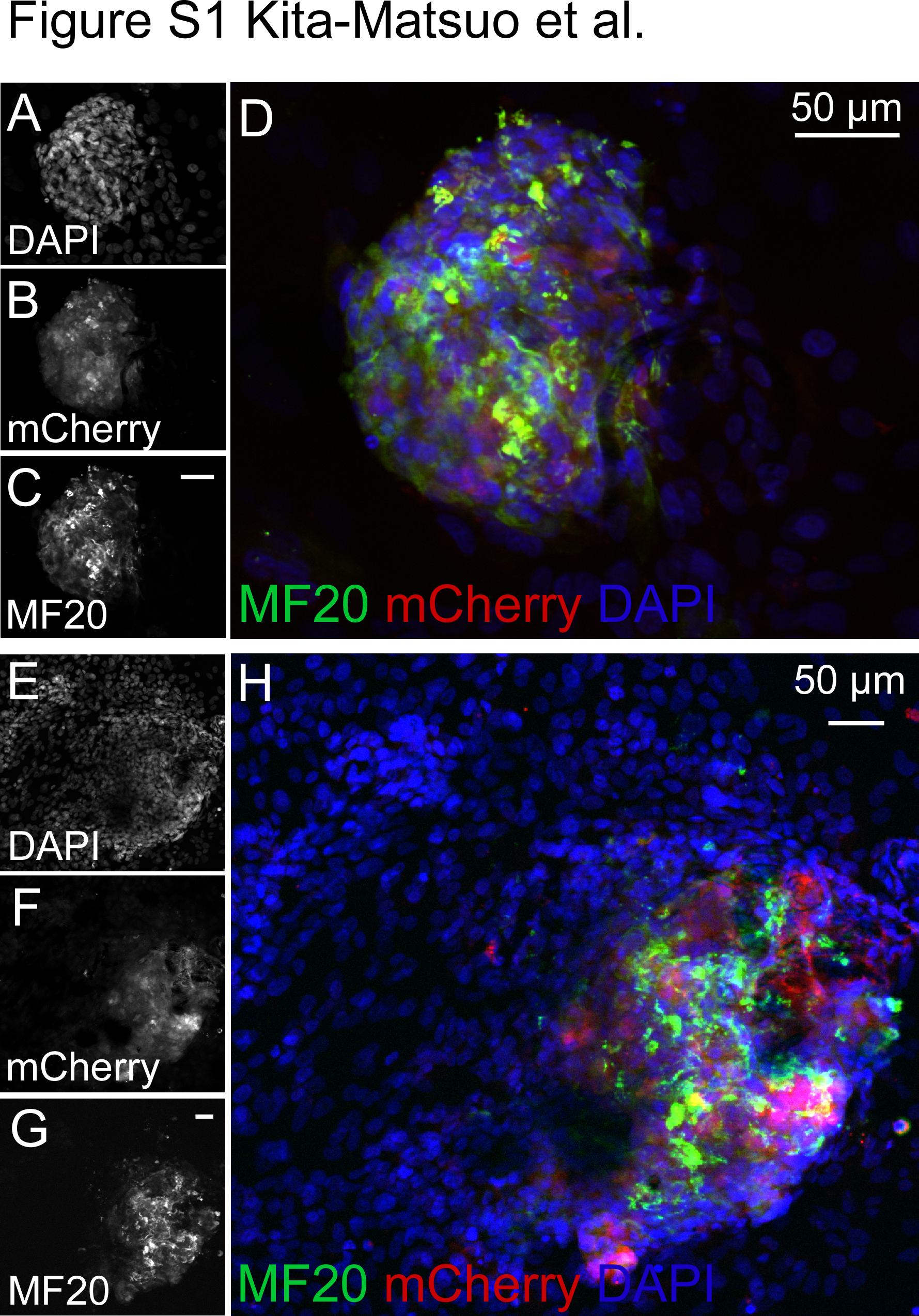

Supplement: Figure S1 — Co-incident fluorescent reporter and myosin expression in EBs derived from engineered hESCs. Examples of differentiating EBs from αMHC-Puror_Rex-Neor, αMHC-mCherry_Rex-Blar, PGK-H2BeGFP hESC line created by G418 and Blasticidin selection. EBs were plated onto gelatin at day 6 of differentiation and processed for immunochemistry at day 10. Micrographs of two EBs imaged at 10× (E–H) and 20× (A–D) showing DAPI (A,E), mCherry (B,F) and MF20 immunostaining (C,G) and merged (D,H) fluorescence. mCherry and MF20 immunostaining fluorescence were coincident in Blasticidin-selected hESCs, reflecting efficacy of the co-selection strategy at removing non-functionally transduced cells. (9.04 MB TIF) [file pone.0005046.s001.tif]

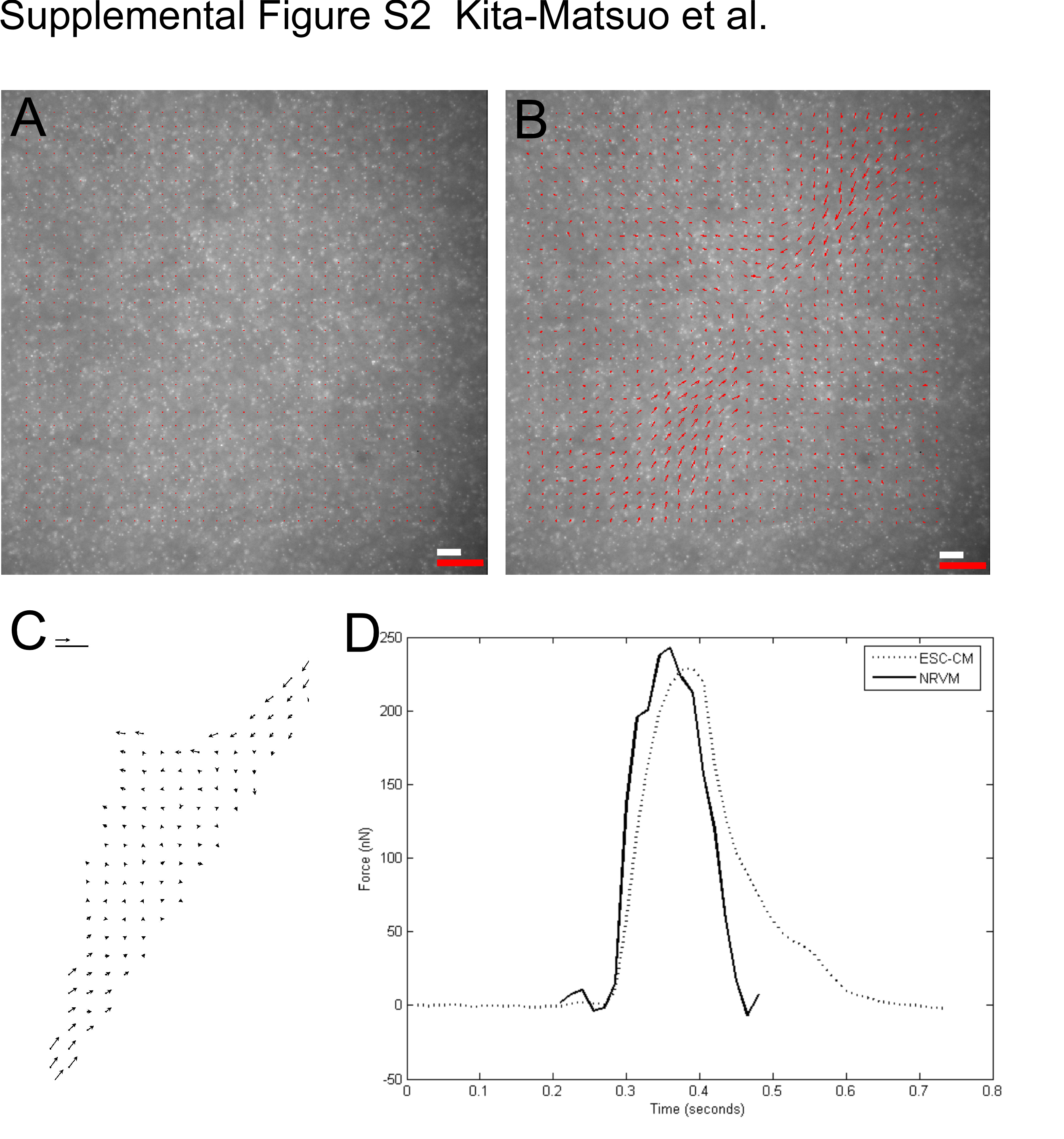

Supplement: Figure S2 — Bead displacement and force generation by day 50 hESC-derived cardiomycytes. Individual hESC-derived cardiomyocytes were deposited onto gelatin functionalized surfaces of polyacrylamide gels incorporating fluorescent beads (see Methods). (A,B) Fluorescent micrographs of bead surface beneath a cardiomyocyte when relaxed (A) and contracted (B). Bead displacements were plotted as deformations or stresses, with displacement vectors as red arrows. Panels are individual frames from the stack shown as Supplemental Movie S5. White scale bars (10 µm) are for the image. Red scale bars (1 µm) represent the scale of the displacement arrows that are expanded 20-fold for visualization. (C) Example of a stress map across the face of the gel during contraction of the hESC-derived cardiomyocyte causing the displacement in (B). The scale bar represents 10 um and scale arrow represents 0.1 nN/µm2. (D) Sample plot of total force over time for hESC-derived cardiomyocyte (dashed) and control neonatal rat ventricular cardiomyocyte (solid) showing similar force generation. (2.19 MB TIF) [file pone.0005046.s002.tif]

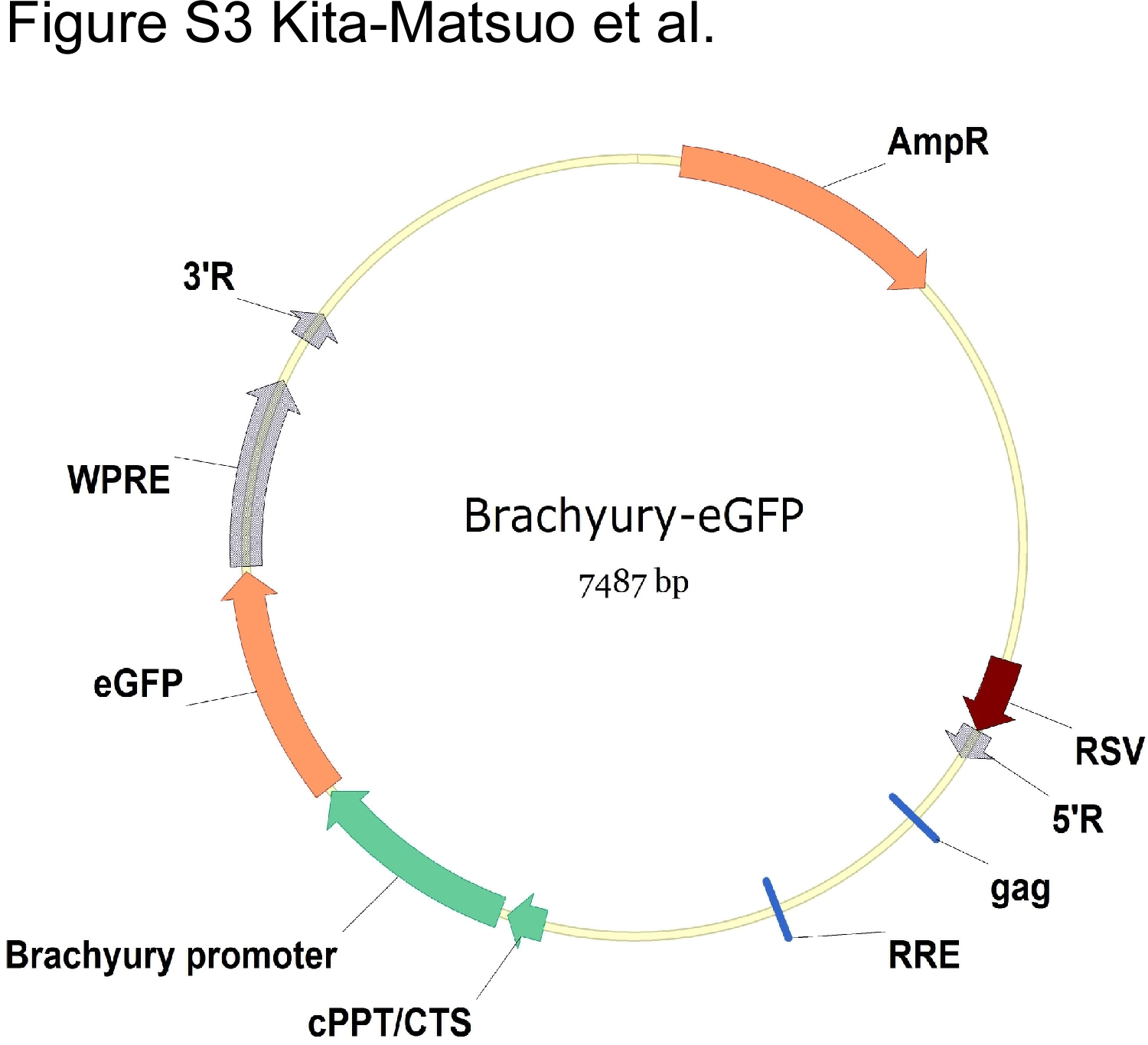

Supplement: Figure S3 — Schematic of T/Brachyury-eGFP. (0.42 MB TIF) [file pone.0005046.s003.tif]

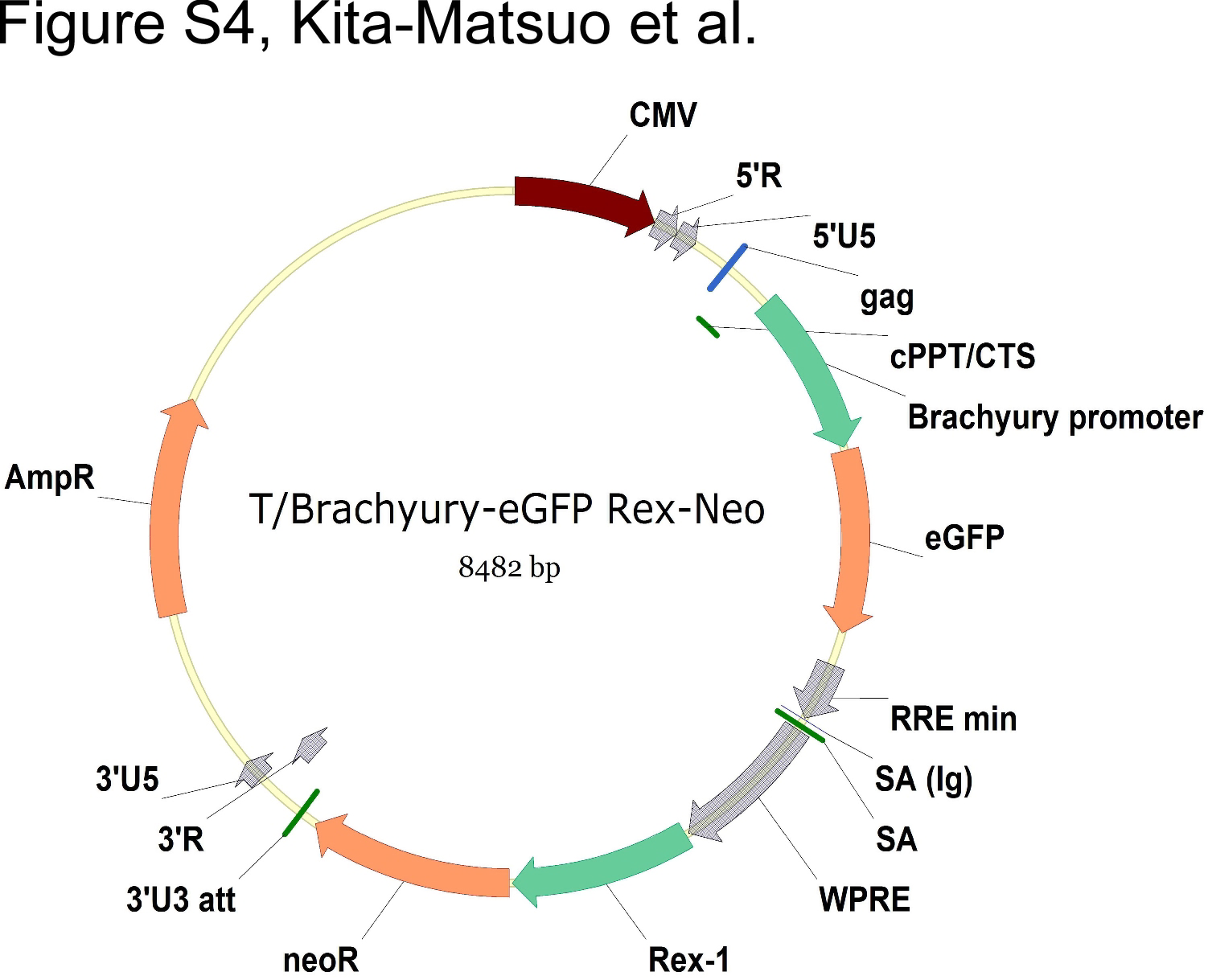

Supplement: Figure S4 — Schematic of T/Brachyury-eGFP_Rex-Neo. (0.45 MB TIF) [file pone.0005046.s004.tif]

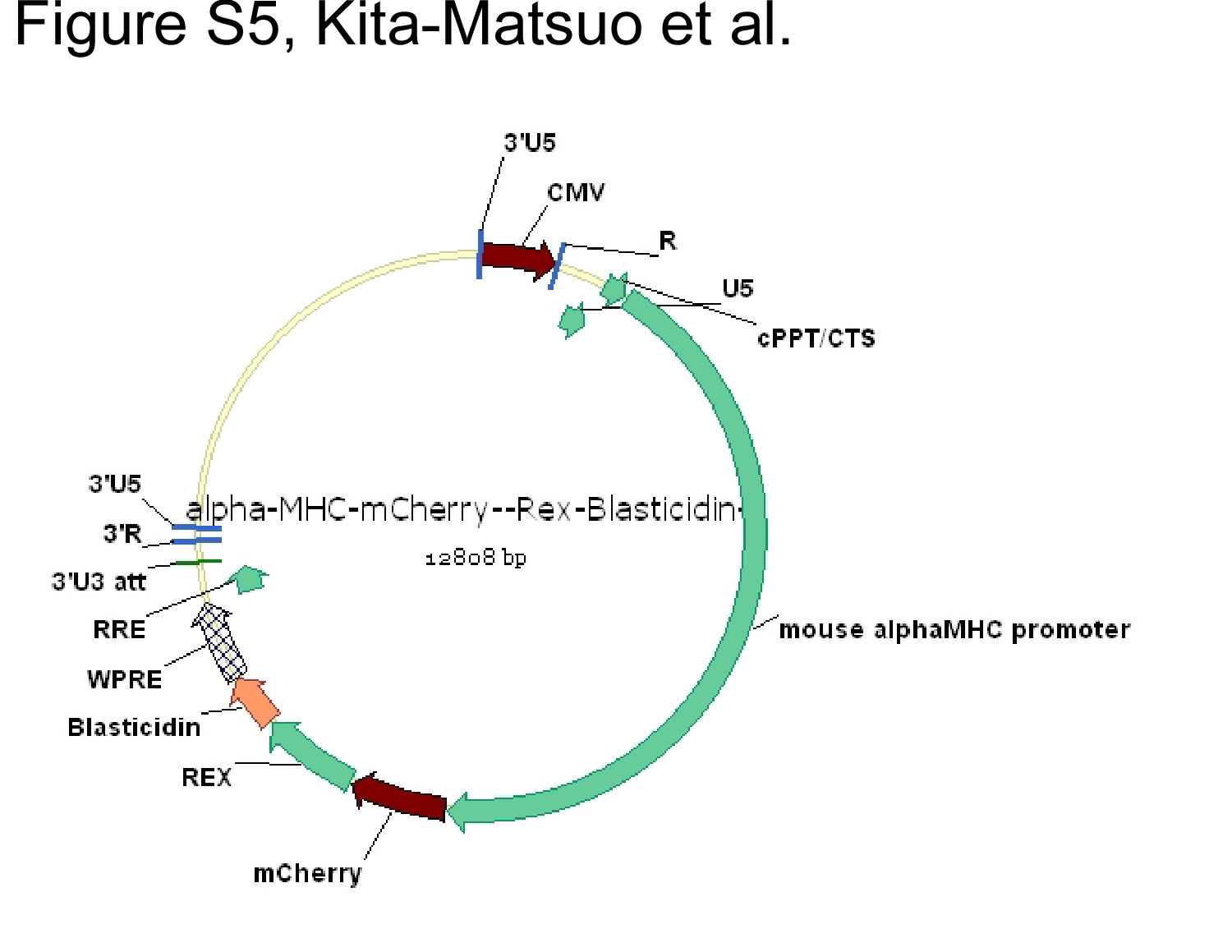

Supplement: Figure S5 — Schematic of αMHC-mCherry_Rex-Blar. (0.28 MB TIF) [file pone.0005046.s005.tif]

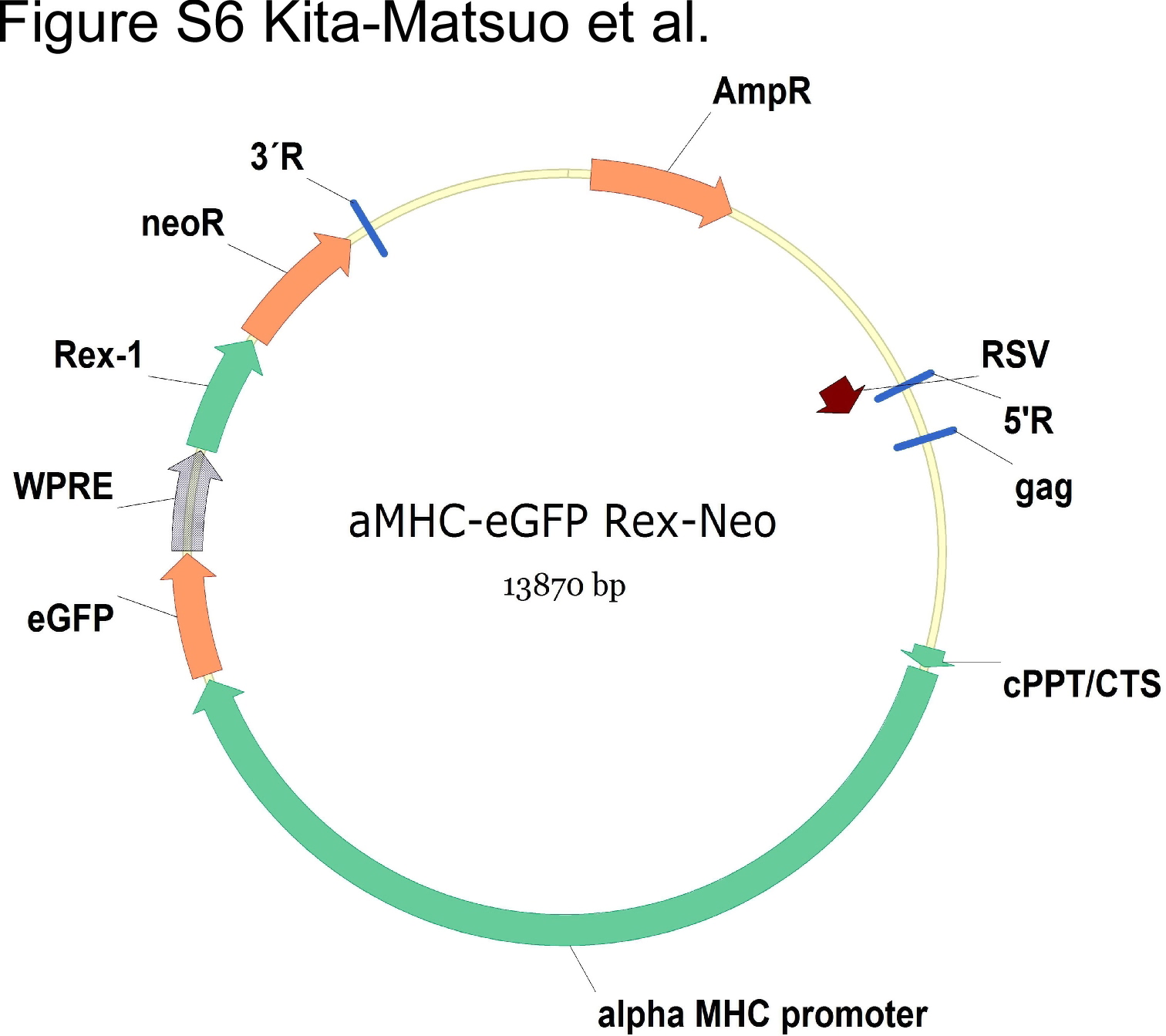

Supplement: Figure S6 — Schematic of αMHC-eGFP_Rex-Neor. (0.45 MB TIF) [file pone.0005046.s006.tif]

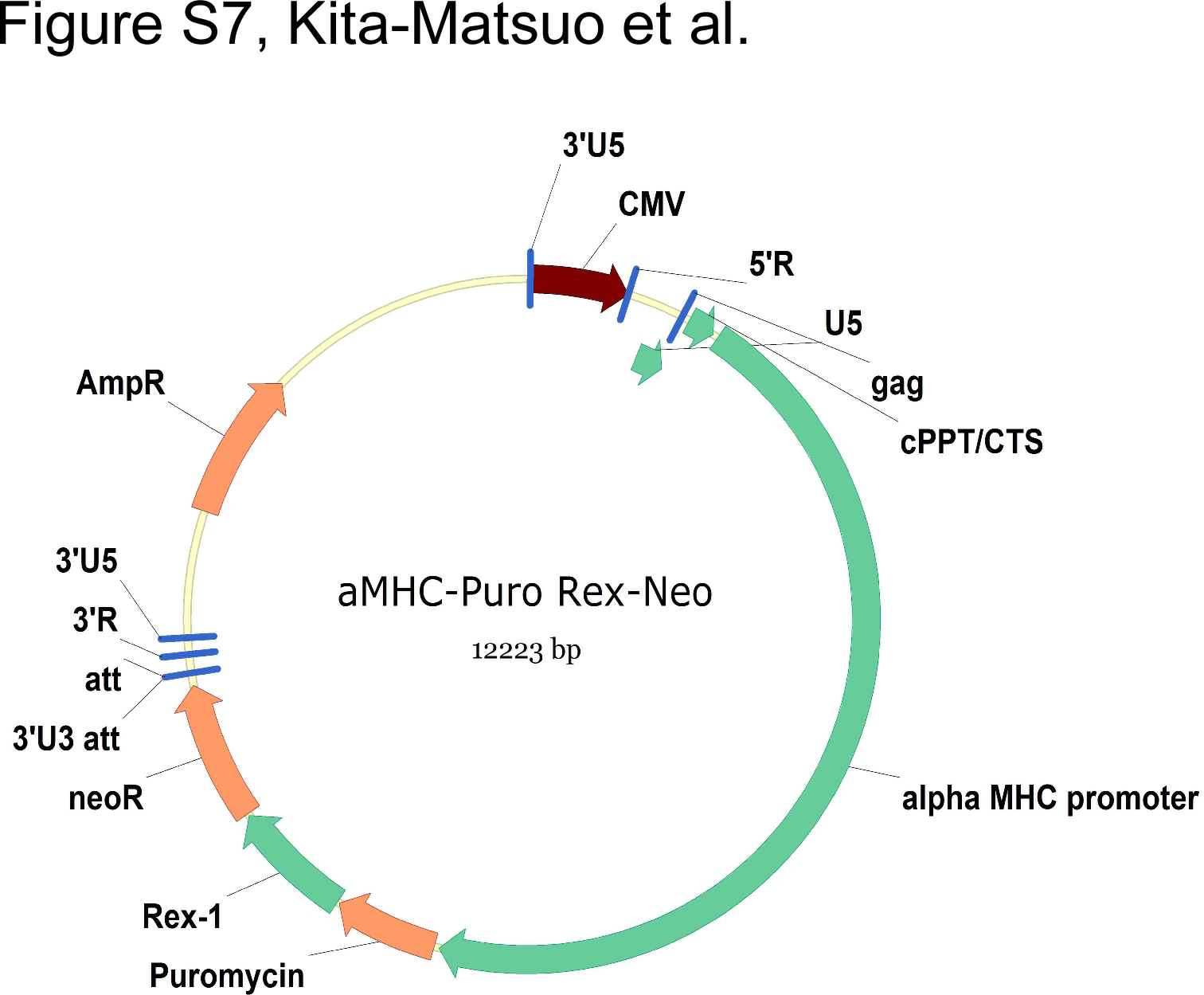

Supplement: Figure S7 — Schematic of αMHC-Puror_Rex-Neor. (0.41 MB TIF) [file pone.0005046.s007.tif]

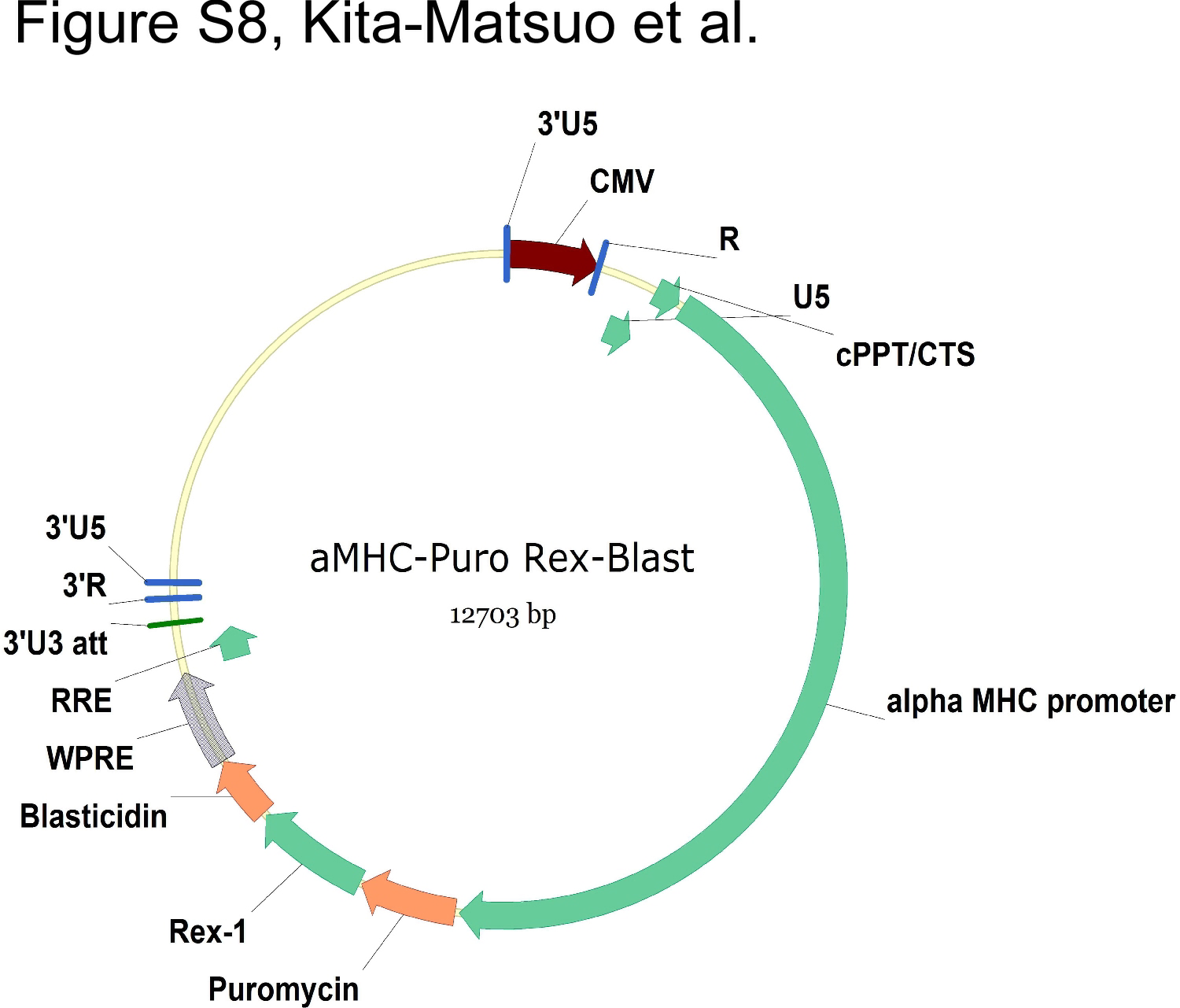

Supplement: Figure S8 — Schematic of αMHC-Puror_Rex-Blar. (0.41 MB TIF) [file pone.0005046.s008.tif]

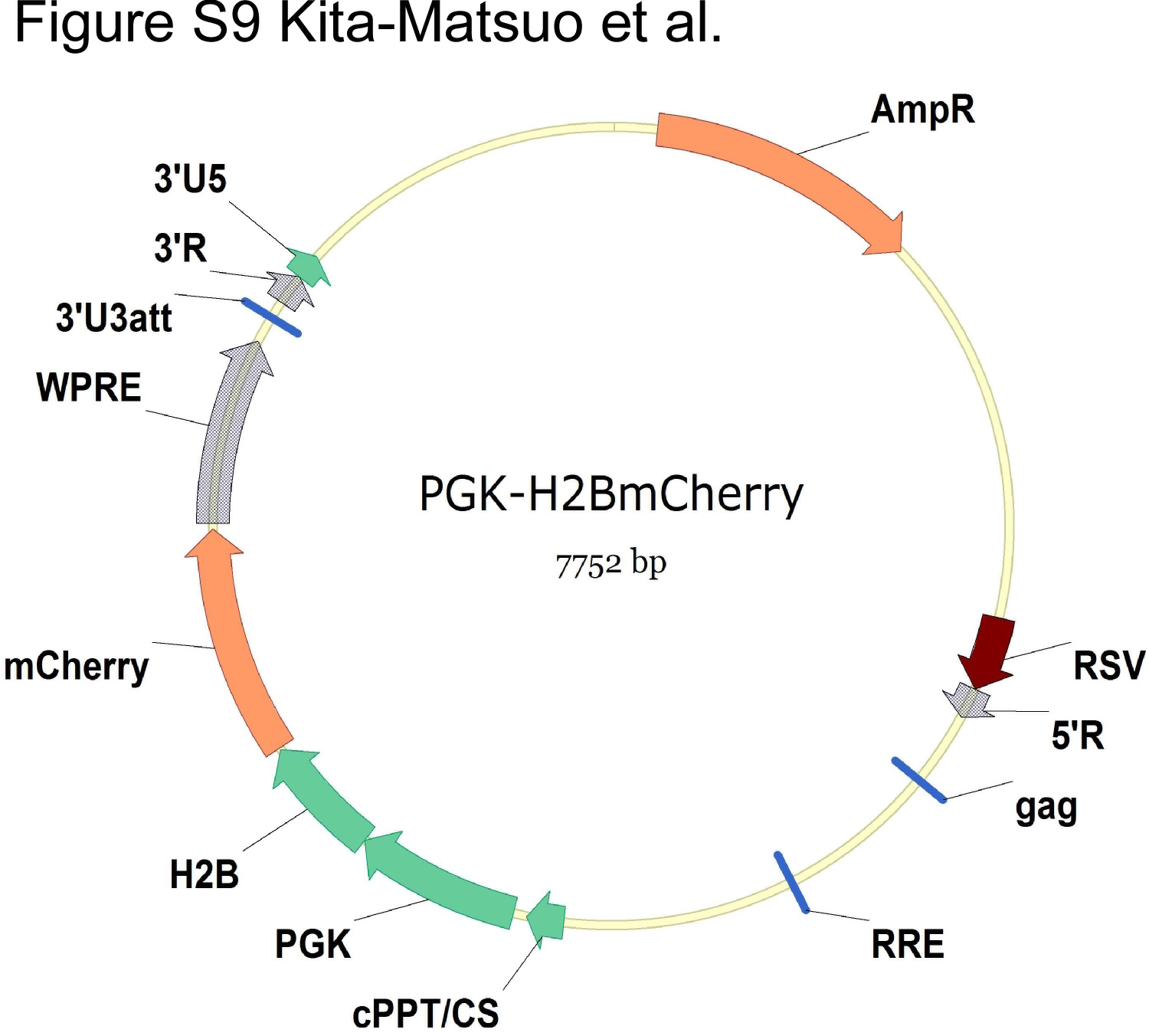

Supplement: Figure S9 — Schematic of PGK-H2BmCherry. (0.55 MB TIF) [file pone.0005046.s009.tif]

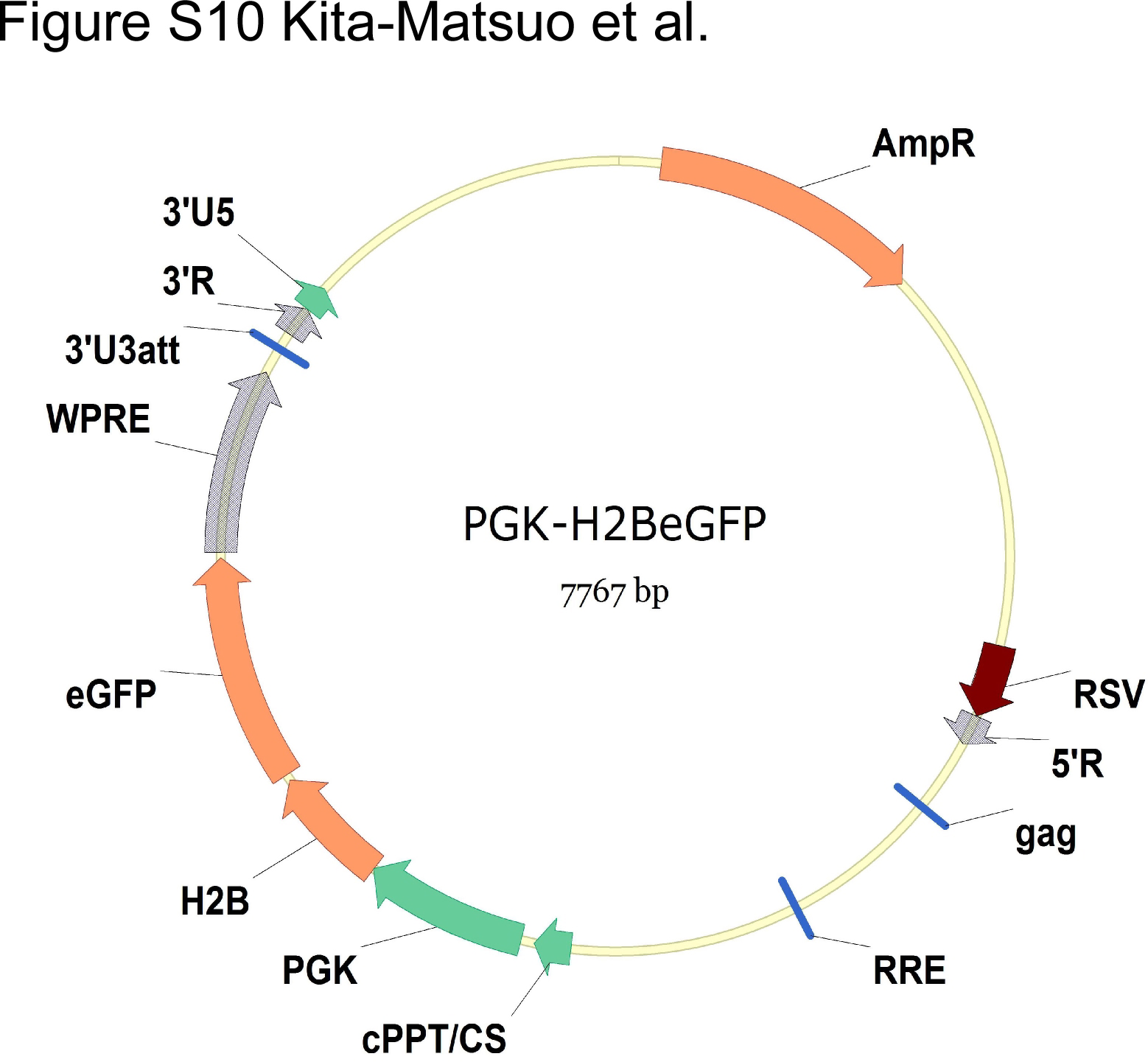

Supplement: Figure S10 — Schematic of PGK-H2BeGFP. (0.50 MB TIF) [file pone.0005046.s010.tif]
